# Resistance and Vulnerability of Honeybee (*Apis mellifera*) Gut Bacteria to Commonly Used Pesticides

**DOI:** 10.3389/fmicb.2021.717990

**Published:** 2021-09-03

**Authors:** Ana Cuesta-Maté, Justinn Renelies-Hamilton, Per Kryger, Annette Bruun Jensen, Veronica M. Sinotte, Michael Poulsen

**Affiliations:** ^1^Section for Ecology and Evolution, Department of Biology, University of Copenhagen, Copenhagen, Denmark; ^2^Entomology and Plant Pathology, Department of Agroecology, Aarhus University, Aarhus, Denmark; ^3^Section for Organismal Biology, Department of Plant and Environmental Sciences, University of Copenhagen, Copenhagen, Denmark

**Keywords:** microbiome, symbiosis, anthropogenic stressor, social insect, neonicotinoid, acaricide

## Abstract

Agricultural and apicultural practices expose honeybees to a range of pesticides that have the potential to negatively affect their physiology, neurobiology, and behavior. Accumulating evidence suggests that these effects extend to the honeybee gut microbiome, which serves important functions for honeybee health. Here we test the potential effects of the pesticides thiacloprid, acetamiprid, and oxalic acid on the gut microbiota of honeybees, first in direct *in vitro* inhibition assays and secondly in an *in vivo* caged bee experiment to test if exposure leads to gut microbiota community changes. We found that thiacloprid did not inhibit the honeybee core gut bacteria *in vitro*, nor did it affect overall community composition or richness *in vivo*. Acetamiprid did also not inhibit bacterial growth *in vitro*, but it did affect community structure within bees. The eight bacterial genera tested showed variable levels of susceptibility to oxalic acid *in vitro*. *In vivo*, treatment with this pesticide reduced amplicon sequence variant (ASV) richness and affected gut microbiome composition, with most marked impact on the common crop bacteria *Lactobacillus kunkeei* and the genus *Bombella*. We conducted network analyses which captured known associations between bacterial members and illustrated the sensitivity of the microbiome to environmental stressors. Our findings point to risks of honeybee exposure to oxalic acid, which has been deemed safe for use in treatment against *Varroa* mites in honeybee colonies, and we advocate for more extensive assessment of the long-term effects that it may have on honeybee health.

## Introduction

The deleterious effects of pesticides on non-target organisms have been a critical area of study in the last decades (Mancini et al., 2019). The vital ecosystem services pollinators provide prioritize assessment of pesticide toxicity in this group, and evidence of pesticide harm to honeybees continues to accumulate (Decourtye et al., 2004a; Gill et al., 2012; Goulson et al., 2015). The western honeybee, *Apis mellifera*, maintains a wide distribution, generalist foraging behavior and pollination competences that make it one of the most important species of pollinators across the world (Hung et al., 2018). Specifically, honeybees play a keystone role in pollination of natural ecosystems and agricultural crops (Goulson et al., 2015; Hristov et al., 2020). However, honeybee populations have declined globally over the past decades, with a continued reduction in colony numbers recorded in the United States, northwestern Europe, and Russia between 1940 and 2010 (Potts et al., 2010; US Department of Agriculture (USDA), 2016, 2021; Brosi et al., 2017), and a striking average annual loss of 30% of colonies reported by northern hemisphere beekeepers over the past decade (Brosi et al., 2017; vanEngelsdorp et al., 2017). A combination of anthropogenic stressors appears to drive honeybee declines (Meeus et al., 2018), including reduced habitats (Naug, 2009; Breeze et al., 2014), reduced genetic diversity (Espregueira Themudo et al., 2020), use of antimicrobials in apiculture (Tian et al., 2012; Li et al., 2017; Raymann et al., 2017), and pesticide application (Boncristiani et al., 2012; Williamson et al., 2014; Johnson, 2015).

Agricultural and apicultural practices expose honeybees to a range of pesticides that can damage their physiology, neurobiology, and behavior, and ultimately may result in colony decline. Two pesticide groups with potentially detrimental effects to honeybee health are insecticides, which are employed within their natural environment, and acaricides, which are applied within colonies. Commonly used neonicotinoid insecticides inhibit nervous system function by antagonizing acetylcholine receptors (Casida and Durkin, 2013). In honeybees, sublethal exposure to nitroguanidine neonicotinoids can alter locomotion (Williamson et al., 2014; Charreton et al., 2015), memory and learning (Decourtye et al., 2004b; Dacher et al., 2005; Shi et al., 2019), consequentially impairing flight ability, navigation (Tosi et al., 2017), and the proboscis extension response to sucrose (El Hassani et al., 2008; Thany et al., 2015). Due to the adverse effects, nitroguanidine neonicotinoids have been banned in several countries (European commission, 2013; Dewar, 2017), while the less toxic cyanoguanidine neonicotinoids remain widely used (Iwasa et al., 2004; Shi et al., 2019). Although cyanoguanidine neonicotinoids, such as thiacloprid and acetamiprid, are considered safer, they can still affect honeybee behavior, memory, and immune functions, and can be lethal at high concentrations (Iwasa et al., 2004; Thany et al., 2015; Brandt et al., 2017; Liu et al., 2020). Honeybees are also frequently exposed to acidic acaricides that are used to treat parasitic *Varroa* mite infestations (Sammataro et al., 2008). Oxalic acid is the most widely used acidic acaricide (Nanetti et al., 2003; Brodschneider et al., 2019) and although its mode of action remains unknown, it is presumed to be safe for bees as it is naturally found in honey (Bogdanov et al., 2002; Moosbeckhofer et al., 2003). Honeybees tolerate treatment concentrations of 3.5% (Rademacher and Harz, 2006), but exposure to higher concentrations can increase mortality and induce behavioral changes, such as reduced nursing efforts or general inactivity (Schneider et al., 2012; Rademacher et al., 2017). Oxalic acid treatment may also alter bee physiology, reduce pH in the digestive tract and the hemolymph (Rademacher et al., 2017), and create permanent lesions in the digestive tract (Martín-Hernández et al., 2007), like necrotic cells (Gregorc and Smodiš Škerl, 2007).

**FIGURE 1:**
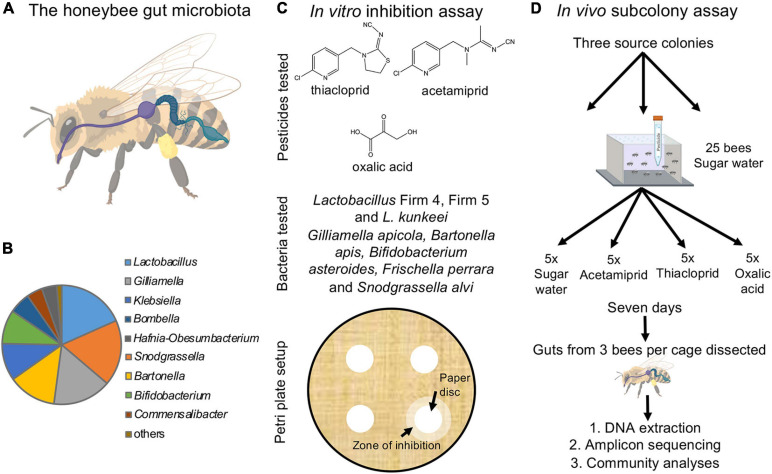
The honeybee gut microbiota and experimental setup. **(A)** The honeybee gut is compartmentalized and includes the crop (purple), midgut (dark blue), and hindgut (soft blue), which is further divided into the ileum and rectum (created and reproduced with permission from Biorender.com). **(B)** The relative abundance of core microbial genera in the honeybee gut microbiota (based on our dataset). **(C)** Overview of the *in vitro* experiment. Lawns of eight core bacteria were used to test the effects of three pesticides inoculated on 1 cm filter paper discs (structures drawn in ChemDraw). Inhibition was scored as the presence/absence of a distinct zone of inhibition forming around the disc. **(D)** Schematic of the *in vivo* experiment. For each of the three colonies, 20 sub-colonies were established each with 25 bees exposed to either of the three pesticides dissolved in sugar water or sugar water alone (control). After 7 days of exposure, three bees per sub-colony were randomly picked, their guts dissected and extracted, and the community composition established using amplicon sequencing.

Until recently, most research on the effect of pesticides on honeybee health focused on the direct effects on the bees, but accumulating evidence suggests that effects extend to the honeybee gut microbiome. The honeybee microbiome is simple and conserved, with 8–10 core bacterial taxa that are omnipresent, regardless of geographical origin (Martinson et al., 2012; Moran et al., 2012; Ellegaard and Engel, 2019; Figures 1A,B). The microbiome modulates immunity against pathogens, partakes in digestion of pollen and in the neutralization of toxins (Engel et al., 2012; Engel and Moran, 2013; Kwong and Moran, 2016; Kešnerová et al., 2017; Raymann and Moran, 2018), promotes host weight and health, and mediates hormonal signaling (Zheng et al., 2016). Adverse effects of certain agricultural compounds on honeybee gut microbes have been documented. The herbicide glyphosate can perturb the absolute and relative abundances of dominant bacterial community members and increase honeybee susceptibility to pathogens (Motta et al., 2018; Blot et al., 2019). Chronic exposure to the highly toxic nitroguanidine neonicotinoids (e.g., imidacloprid and thiamethoxam) in laboratory settings can induce changes in gut bacteria community composition of healthy honeybees (Rouzé et al., 2019). However, these results have not been found when bees are returned to the hive after exposure (Raymann et al., 2018b). Thiacloprid, a cyanoguanidine neonicotinoid, potentially invokes dysbiosis of the gut microbiome (Liu et al., 2020), and although lower field-level concentrations may not alter colony performance, they reduce immune expression against pathogens (Siede et al., 2017). The effects of other commonly used cyanoguanidine neonicotinoids, such as acetamiprid, have yet to be investigated. Pesticides and antimicrobials used in apiculture to mitigate parasite and pathogen infections can also impact the honeybee gut microbiota (Li et al., 2017; Raymann et al., 2017, 2018a). Acaricides such as coumaphos and tau-fluvalinate can influence the structure of the bacterial community in the gut (Kakumanu et al., 2016). Oxalic acid has antibacterial activity, including against *Lactobacillus* strains isolated from honeybees (Diaz et al., 2019), yet inhibition of other key core taxa and alteration of the gut microbial community have not been evaluated. Thus, our knowledge remains sparse despite the potential adverse effects of commonly used pesticides on honeybee gut microbes.

In this study, we address the potential effects of the pesticides acetamiprid, thiacloprid, and oxalic acid on the gut microbiota associated with honeybees. These compounds are still used on a global scale (Adjlane et al., 2016; Amulen et al., 2017; US Environmental Protection Agency Office of Pesticide, 2020; Begna and Jung, 2021), although thiacloprid was recently withdrawn from approval in Europe (European Commission, 2020; European Food Safety Authority (EFSA), 2020). Oxalic acid is a widely used treatment against *Varroa* (Rademacher et al., 2017). These pesticides have been deemed safe for bees, but insight into how they may affect honeybee gut bacterial communities is necessary to obtain a more complete risk assessment. We first performed *in vitro* assays to elucidate the potential direct negative effects of these compounds on main core bacterial members of the honeybee gut microbiome (Figure 1C). Subsequently, we performed an *in vivo* experiment to investigate if the indications from the *in vitro* experiment translated to community effects on the microbiome and changes in honeybee health (Figure 1D).

## Materials and Methods

### *In vitro* Assay of Gut Bacteria Susceptibility to Pesticides

To test for effects of the acetamiprid, thiacloprid, and oxalic acid on the growth of honeybee gut microbes, we obtained 15 strains from eight core gut bacteria of the western honeybee (*A. mellifera*) (Figure 1C; Moran et al., 2012; Bonilla-Rosso and Engel, 2018; Ellegaard and Engel, 2019). Specifically, we obtained two strains of *Lactobacillus* Firm-4 (codes ESL0291 and ESL0292), two strains of *Lactobacillus* Firm-5 (codes ESL0183 and ESL0184), two strains of *Lactobacillus kunkeei* (ESL0216 and ESL0219), two strains of *Bifidobacterium asteroides* (ESL0199 and ESL0200), two strains of *Gilliamella apicola* (ESL0171 and ESL0172), two strains of *Snodgrassella alvi* (ESL0145 and ESL0252), two strains of *Bartonella apis* (ESL0058 and ESL0059) and one strain of *Frischella perrara* (ESL0215). *Lactobacillus* and *Bifidobacterium* strains were maintained on de Man, Rogosa and Sharpe agar (MRSA) and cultured anaerobically; *Gilliamella, Snodgrassella*, *Bartonella*, and *Frischella* strains were maintained on Columbia Blood agar (CBA) +5% blood medium. The former three genera were kept at 5% CO_2_, while the latter was maintained under anaerobic conditions. All strains were kept at 34°C (cf. Kešnerová et al., 2016, 2017).

For standardized growth, five biological replicates of each strain were grown in liquid media, de Man, Rogosa and Sharpe (MRS) for *Lactobacillus* and *Bifidobacterium* strains, Columbia Blood (CB) for *Gilliamella*, *Snodgrassella, Bartonella*, and *Frischella* strains), and once OD600 reached 0.5 (after 1 day), 100 μl of each culture was plated in solid media in two 90 mm petri dishes to generate a bacterial lawn. Then, adapting from Balouiri et al., 2016, once the inoculum dried on the plate, four 5 mm sterilized paper filter discs were placed on the surface of the plate. The paper disc received 5 μl of the compound solution, and 5 μl of sterile water in the case of the controls. The concentrations chosen for each of these compounds were based on their solubility in water: acetamiprid (4.2 mg/ml), thiacloprid (0.1 mg/ml), and oxalic acid (95 mg/ml) to ensure that we tested effects of maximum concentrations, although these are likely to be far higher than those encountered in the field. This trial revealed that the bacteria were only sensitive to oxalic acid, and we consequently tested its effects at concentrations 0.095, 0.95, 9.5 mg/ml, which are conceivably more common doses for microbes to be exposed to than the very high maximum dose (Charrière and Imdorf, 2002; Schneider et al., 2012). Each strain was grown either without (controls) or with (treatments) a compound present in replicates of five. Presence/absence of inhibition was scored 2 days after exposure (Figure 1C).

### *In vivo* Assessment of Pesticide Consumption on Gut Microbiota

#### Honeybee Collection and Sub-Colony Set-Up

Honeybees (*A. mellifera*) from three different colonies were obtained from the Department of Agroecology, Aarhus University, Research Centre Flakkebjerg in Slagelse, Denmark on the 7th of September, 2020. The colonies were opened, and the queen was located. Once found, bees were sampled from frames without the queen so that the colony would keep producing brood. Drones were avoided. Adult workers were picked from the bottom of the frame in an effort to avoid foragers and older bees. This implies that we most likely predominantly sampled younger bees working within the hive, with more stable gut microbiomes than foragers (Jones et al., 2018). Although it would have been ideal to sample comparably aged bees across the three nests, the consistent responses of the three colonies (see section “Results”) suggest that differences in age are unlikely to have impacted our findings. The bees were then placed in Styrofoam boxes for transport to the University of Copenhagen. The three queens from the colonies were half-sisters.

At the University of Copenhagen, bees were anesthetized with CO_2_ so that they could be separated into experimental groups. The Styrofoam boxes were put in plastic bags that were filled with CO_2_ and, once the bees were inactive, they were sorted into their respective experimental groups. Bees from each colony were divided in five replicates (25 bees per replicate) for each experimental group and housed in rectangular plastic boxes of 18 × 11 × 6 cm, similar to previous work (Evans et al., 2009; Huang et al., 2014; Figure 1D). Each of the boxes had sixteen 2 mm diameter holes in the lid for ventilation, and a larger hole was drilled in the lid to allow placing a 15 ml Falcon tube for feeding. The Falcon tube contained 12 ml 50% sucrose diet without pesticides for controls and with one of the three pesticides for treatment sub-colonies. Each Falcon tube had three holes in the bottom from which the bees were able to feed (Huang et al., 2014; Figure 1D).

Compound concentrations in the sugar water provided to treatment sub-colonies were based on previous work. Thany et al. (2015) tested 0.1 μg acetamiprid/bee and El Hassani et al. (2008) tested 0.5 μg/bee. We decided to follow the former, because it had the concentration that was closest to what we expect bees to encounter in the environment (134 ppb, cf. Mullin et al., 2010). For the calculations, the onetime dose was multiplied by the number of bees per sub-colony (25) and the number of days that the experiment lasted (7). For thiacloprid, Mullin et al. (2010) detected traces of thiacloprid in 2% of pollen samples, at levels of 7.8 PPB. Zaworra et al. (2019) registered an IC50 (inhibitory concentration for half the replicates) of 0.19 μg. Tison et al. (2017) found that concentrations of 0.5–50 μg/ml did not increase mortality, but the highest did alter memory, we therefore used the lowest concentration. Schneider et al. (2012) used a 3.5% oxalic acid treatment (3500 μg/ml), which they reported to be one of the most common concentration beekeepers use in beehives (Charrière and Imdorf, 2002). Rademacher et al. (2017) used a concentration of 50 μg/bee, considering this to be more representative of what was found in bee colonies (acknowledging the probable accumulation within colonies). Therefore, we used the second value and calculated the concentration in the same manner as for acetamiprid. Based on this, we provided bees access to sugar water with a concentration of 3.5 μg/ml of acetamiprid, 0.05 μg/ml of thiacloprid, or 1750 μg/ml oxalic acid. Although concentrations used thus vary greatly between compounds, they were chosen to best reflect ecological relevance.

After sub-colonies were setup, they were kept at room temperature with dim light. The sub-colonies were checked daily to count the number of dead bees and to check if sugar water was consumed or not. We did not remove dead bees during the experiment to minimize disturbance and to reduce the risk of escapees. The approximate volume of sugar water consumed was recorded on days one through four, after which the volume decreased such that it was not easily discernable and thus could not be recorded. The Falcon tube was refilled as needed with each treatment. The experiment lasted for 7 days, after which dead and live bees were collected and frozen separately. The gut dissections following the experiment were performed only on the live bees.

### Gut Dissection, DNA Extraction, and 16S rRNA Amplicon Sequencing

Gut dissections were performed in sterile conditions on up to three bees per sub-colony, for a total of 146 bees (*n*_control_ = 36, *n*_acetemiprid_ = 39, *n*_thiacloprid_ = 39, and *n*_oxalic acid_ = 32). The dissection area was sterilized with 70% ethanol and the dissection materials with 96% ethanol and a flame, and the dissections were performed in the presence of a Bunsen burner (cf. Carreck et al., 2013; Engel et al., 2013). The bee cuticle was sterilized prior to dissection by soaking the bee in a 1% aqueous solution of bleach for 3 min and then rinsing it in sterile purified water for three times 30 s (Binetruy et al., 2019). The bee gut was obtained by gently pulling the sting out of the abdomen, and the whole gut came attached to it. The gut was placed in a screw top 2 ml Eppendorf tube with 50 μl sterile PBS and frozen at −20°C for later DNA extraction. In addition to the experimental bees, we included 12 bees from each colony that had been frozen immediately after collection (termed field controls). These field controls turned out to differ in community composition from our experimental controls, so we excluded them from the main manuscript analyses, but include them in a set of Supplementary Text, Supplementary Figures 1–3, 5–7, and Supplementary Tables 1, 2, 9.

For DNA extractions, we used the Qiagen Blood and Tissue kit (Qiagen, Hilden, Germany), following the manufacturer’s protocol, but modified based on Engel et al. (2013). For the tissue lysis, we used sterile 7 mm stainless beads and added 250 μl of 0.1 mm sterile crystal beads, running the bead beater at 30 Hz twice for 1 and 2 min, respectively. A total of 180 μl ATL buffer and 20 μl proteinase K were added to each sample, vortexed and incubated at 56°C for 3 h on a rotor. After incubation, 4 μl RNase A was added and the sample was incubated for 15–20 min. The remainder of the protocol was as per the manufacture’s protocol. The elution volume was 100 μl of buffer AE, and it was passed through the column twice in a joint elute.

Diagnostic PCR was conducted to confirm the presence of sufficient bacterial DNA in 143 samples. We used primers for the V4 region of the 16S rRNA gene (′V4.SA504: 5′-AAT GATACGGCGACCACCGAGATCTACACCTGCGTGTTATGG TAATTGTGTGCCAGCMGCCGCGGTAA-3′ and ′V4.SB711: 5′-CAAGCAGAAGACGGCATACGAGATTCAGCGTTAGTCA GTCAGCCGGACTACHVGGGTWTCTAAT-3′). PCR conditions were 94°C for 4 min, 35 cycles of 94°C for 30 s, 56°C for 30 s, and 72°C for 30 s, and a final extension step of 74°C for 4 min. Amplifications were confirmed on a 2% agarose gel. Three negative controls without guts and a positive control with a cellular mock community standard (Zymobiomics, Nordic BioSite ApS, Copenhagen) were included in the DNA extraction, and three additional negative control were added during library preparation and sequencing. DNA was sent to the University of Michigan’s Microbiome Core^1^ for paired-end 250 bp 16S rRNA Illumina MiSeq amplicon sequencing with ′V4.SA504 and ′V4.SB711 primers.

### Statistical Analyses

All statistical analyses were performed in R studio v. 4.0.3 R Core Team (2020).

For the bacterial inhibition assay, a generalized linear mixed-effects model (GLMM) with family binomial was used to address the effect of oxalic acid concentration on bacterial inhibition. Concentration was included as a fixed effect and bacterial strains as a random slope. Model reduction with subsequent ANOVA comparison was performed to address the effect of concentration on inhibition.

To analyze the mortality data (Supplementary Table 3), we used Cox proportional hazard regression models and likelihood ratio (LR) test statistics, employing the R package survival and the function coxph (version 3.2-7; Therneau, 2021). Treatment was included as a fixed effect, where treatments were compared to the control, and colony was included as a stratified fixed effect. Additionally, we created models to assess colony-level responses to treatment with treatment a fixed effect. For all models, the proportional hazard assumptions and the Cox-Snell residuals were tested according to Mills (2012); our models met both assumptions.

We used a GLMM with family binomial to assess if the type of pesticide affected whether or not the bees consumed sugar water. Treatment, colony, and day were inserted as fixed effects and sub-colony as a random slope. We then assessed the average volume of pesticide or control solution consumed by each bee over days one through four by dividing the daily consumption of the sub-colony by the number of bees alive in the sub-colony that day. We then used a general linear model (LM) to test the fixed effects of treatment, day, and colony on the volume of control or pesticide solution consumed. The minimal model was determined with backwards model reduction and met assumptions of normality and homoscedasticity. In both models, subsequent model reduction and ANOVA comparisons were performed to address the significance of the fixed effects on consumption and volume consumed.

For the 16S rRNA gene amplicon sequencing analysis, we used the dada2 pipeline (v.1.12.1; Callahan et al., 2016) and performed downstream analysis with default parameters with the following adjustments: we set the truncLen parameter in filterAndTrim to c(240,220), trimleft (6) and maxEE to c(2,3). We obtained 610 amplicon sequence variants (ASVs) after quality filtering and removing chimeric reads. The merged sequences were assigned to taxonomic ranks using the assignTaxonomy function in the dada2 package in R using the SILVA database release 138.1 (Quast et al., 2013). Since negative controls included core honeybee gut microbiome taxa, an abundance threshold was chosen to include genera in our study, while avoiding contaminants (cf. Kešnerová et al., 2017; Raymann et al., 2017). Only genera with an abundance >0.08% across the whole dataset were retained for further analysis, resulting in 15 genera and 203 ASVs. By removing non-abundant genera, we reduce the presence of putative contaminants, while keeping 98.7% of the total number of original reads, including genera that are part of the honeybee gut core microbiota and present in low levels in negative controls. The cellular mock community validated the detection of all eight expected taxa and allowed for an estimation of the random error in our community abundances, averaging 0.07% of the abundance of any given taxon.

Richness was estimated as the number of ASVs using the function estimate_richness in the R package phyloseq (v.1.34.0; McMurdie and Holmes, 2013). To test for statistical differences in richness between treatments and colony origin, a LM was used, where the log of the observed number of ASVs per treatment was the response variable. Both treatment and colony were included as fixed effects, and the model included their interaction. Pairwise comparisons between treatments were preformed using Tukey honestly significant difference (HSD) test, after confirming that the model assumptions were met using shapiro.test (v.0.9-38; Royston, 1982) and bptest (Breusch and Pagan, 1979) from the lmtest package (v.0.9-38; Zeileis and Hothorn, 2002). Beta diversity metrics were calculated based on Bray–Curtis and Jaccard distances, using the vegdist function from the vegan package in R (v.2.5-7; Oksanen et al., 2018). Additionally, Unifrac distances were also calculated using the UniFrac function. The differences between controls and each treatment were separately tested using multivariate PERMANOVAs (adonis function in the vegan package), together with a colony fixed effect and the interaction between colony and treatment.

ALDEx2 (v.1.22.0; Fernandes et al., 2013) was used to analyze differentially abundant genera between controls and each pesticide treatment group. The same was done to detect differentially abundant ASVs. In each case, we controlled for colony by adding it as a fixed effect.

Lastly, as previous work has reported syntrophic relationships among honeybee gut symbionts (e.g., Kwong and Moran, 2016; Kešnerová et al., 2017), we performed network analyses using Bray–Curtis distance in the plot_net function in phyloseq for each treatment separately to test if associations in the control group were maintained with different pesticide treatments. We performed the analysis across all colonies to secure a sample size that allowed for a robust analysis. Distances below 0.5 are reported as associations.

## Results

### Only Oxalic Acid Inhibited Bacterial Growth *in vitro*

**FIGURE 2:**
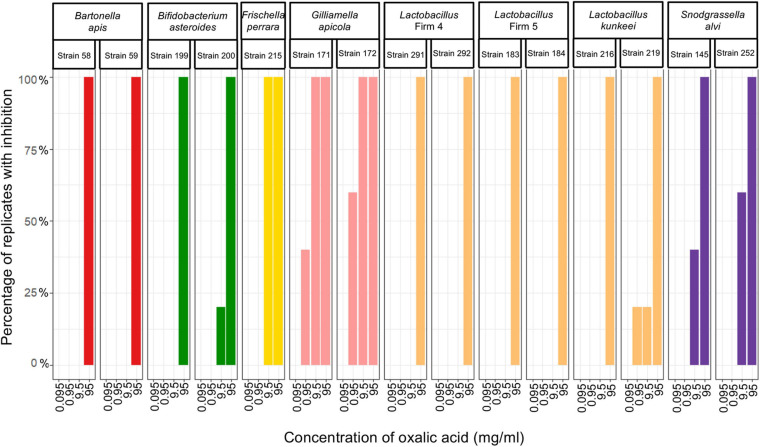
Honeybee bacteria isolates inhibited by oxalic acid. The percentage of cultures that were inhibited after exposure to each of the four concentrations of oxalic acid (*n* = 5 for all cultures, except for *Lactobacillus* Firm-4, strain 291, where *n* = 4 for 95 mg/ml and *n* = 3 for 9.5 and 0.95 mg/ml).

We evaluated the proportion of plates with honeybee gut bacteria that were inhibited across the specified concentrations of three pesticides and found that all strains were resistant to acetamiprid and thiacloprid, but sensitive to oxalic acid (Figure 2). *B. apis* and *Lactobacillus* Firm-5 were only inhibited at the highest concentration (95 mg/ml), which is unlikely to be encountered in the field. Bacteria susceptible to concentrations relevant to acaricide application and bioaccumulation included *S. alvi*, *F. perrara* and *B. asteroides*, which were inhibited at 9.5 mg/ml, and *Lactobacillus* Firm-4, *L. kunkeei*, and *G. apicola*, which were most sensitive and inhibited already at 0.95 mg/ml; although, the latter for only one of the five test plates (Figure 2). The concentration of oxalic acid had a significant effect on inhibition (overall GLMM: *F*_2_,_220_ = 25.13; concentration: χ^2^ = 151.8, df = 3, *p* < 0.0001).

### Pesticide Effects on Honeybee Mortality and Liquid Consumption

**FIGURE 3:**
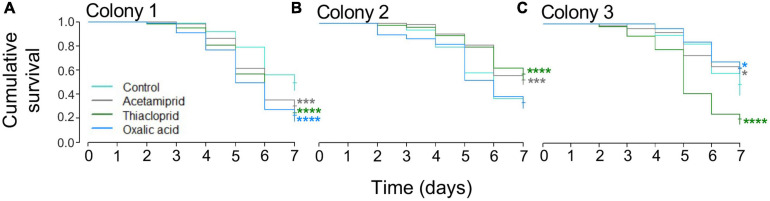
Mortality across colonies and treatment in the *in vivo* caged bee experiment. **(A)** For colony 1, acetamiprid, thiacloprid, and oxalic acid reduced survival when compared to the control. **(B)** For colony 2, acetamiprid and thiacloprid increased survival when compared to the control. **(C)** For colony 3, both acetamiprid and oxalic acid increased survival, while oxalic acid increased mortality. *p < 0.05, ***p < 0.001, and ****p < 0.0001 (Table 1).

Mortality was high across treatments, including in controls (Figure 3). The Cox proportional hazard regression model test indicated significant effects of treatment (overall model LR test: LR = 23.6, df = 3, *p* < 0.0001). The hazard ratios (HR) were calculated for each treatment, a hazard ratio >1 means that the treatment increased mortality compared to the control, while values <1 indicate that treatment decreased mortality compared to controls. Exposure to thiacloprid significantly increased bee mortality (Wald statistic: *z* = −1.04, *p* = 0.0007, HR = 1.357; Supplementary Figure 4), as did oxalic acid (*z* = 3.36, *p* = 0.0332, HR = 1.217; Supplementary Figure 4), but this was not the case for acetamiprid (*z* = 2.13, *p* = 0.2974, HR = 0.905) (Table 1 and Supplementary Figure 4). For the models on individual colonies, treatment had an effect in colony 1 (LR = 32.58, df = 3, *p* < 0.0001; Figure 3A), colony 2 (LR = 31.43, df = 3, *p* < 0.0001; Figure 3B), and colony 3 (LR = 81.45, df = 3, *p* < 0.0001; Figure 3C). In colony 1, all three pesticides increased mortality, in colony 2, acetamiprid and thiacloprid had less mortality than controls, and in colony 3, acetamiprid and oxalic acid reduced mortality and thiacloprid increased mortality (Figure 3 and Table 1).

**TABLE 1:** Effect of treatment on mortality within the three colonies compared to controls, based on Cox proportional hazards regressions.

Colony	Treatment (compared to control)	Hazard ratio (HR)	Wald statistic (*z*)	*p*
1	Acetamiprid	1.699	3.367	0.0007
	Thiacloprid	1.971	4.353	<0.0001
	Oxalic acid	2.236	5.201	<0.0001
2	Acetamiprid	0.565	–3.465	0.0005
	Thiacloprid	0.511	–3.972	<0.0001
	Oxalic acid	1.059	0.383	0.7019
3	Acetamiprid	0.635	–2.466	0.0136
	Thiacloprid	2.349	5.604	<0.0001
	Oxalic acid	0.631	–2.552	0.0107

*HR > 1 indicates that the treatment increased mortality compared to the control, while HR < 1 means that the treatment decreased mortality compared to the control.*

Treatment significantly impacted the presence/absence of consumption (overall GLMM: *F*_3_,_415_ = 8.07; treatment: χ^2^ = 38.19, df = 3, *p* < 0.0001) (Supplementary Table 4), while day and colony did not (day: χ^2^ = 0.12, df = 1, *p* = 0.7307; colony: χ^2^ = 1.62, df = 1, *p* = 0.2035). Across all experimental days, the total number of events (consumption or no consumption) was 105. Of these accounts, consumption was lowest for oxalic acid (78 accounts), followed by acetamiprid (96 accounts), controls (101 accounts), and thiacloprid (103 accounts). Treatment, day, and colony all significantly impacted consumption volume on the first 4 days of treatment (overall LM: *F*_6_,_177_ = 12.51, *p* < 0.0001). Treatment had a significant effect (LM: *F*_3_,_177_ = 5.16, *p* = 0.0019), where when compared to the control, thiacloprid significantly increased consumption (*p* = 0.0464), while there was no significant difference for acetamiprid (*p* = 0.5785) or oxalic acid (*p* = 0.0773). Consumption significantly reduced over days one through four (LM: *F*_1_,_177_ = 38.81, *p* < 0.0001; Supplementary Tables 5, 6), and colony had a significant effect (*F*_2_,_177_ = 7.65, *p* = 0.0006; Supplementary Tables 5, 6).

### Pesticides Affect Richness and Beta Diversity of the Honeybee Gut Bacterial Community

**FIGURE 4:**
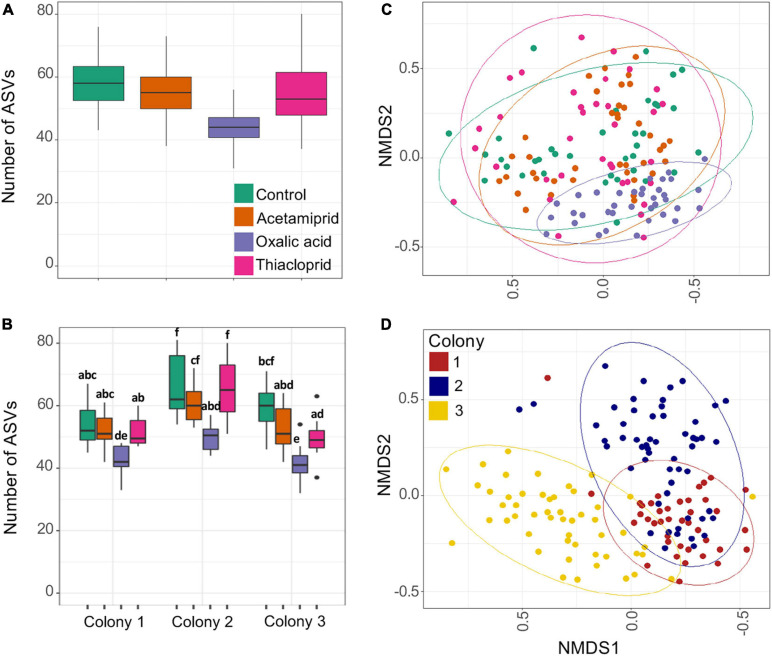
Gut microbiome richness and beta diversity estimates of pesticide treated and control honeybees. **(A)** ASV richness across treatments. **(B)** ASV richness across colonies and treatments. Boxplots represent the first and third quartiles of the number of ASVs observed per treatment, the horizontal line represents the median, whiskers extend 1.5 interquartile ranges and dots represent outliers. In panel **(B)**, the letters represent the statistical dissimilarities across groups according to Tukey HSD test (*p* < 0.05). **(C)** Non-metric multidimensional scaling (NMDS) analysis of the samples based on Bray–Curtis distances; plot ordination based on treatment. **(D)** Non-metric multidimensional scaling (NMDS) analysis of the samples based on Bray–Curtis distances; plot ordination based on colony. There is a clear clustering of the samples in the ordination plot based on colonies, which is supported by the PERMANOVA test.

We obtained a total of 4,710,340 clean MiSeq reads of the 16S rRNA gene (average 27,546/sample) and rarefaction curves supported sufficient coverage to pursue diversity analyses (Supplementary Figure 5). When compared to the control group, ASV richness was significantly affected by the consumption of oxalic acid, which reduced richness by 25.7% (*p* < 0.0001). Treatment had an effect on ASV richness (Figure 4A and Supplementary Table 7; LM: *F*_3_,_134_ = 34.31), but acetamiprid (*p* = 0.0522) and thiacloprid treatments (*p* = 0.2602) did not differ from controls. There were also significant differences in microbial richness between colonies (*F*_2_,_134_ = 45.07; *p* < 0.0001; Figure 4B and Supplementary Table 8), but the effect of pesticides was the same on all colonies, as evident from the non-significant interaction between treatment and colony (*F*_6_,_134_ = 1.297; *p* = 0.2629).

Beta diversity differences were only to a small extent driven by pesticide treatment (Figure 4C) but affected more by colony (Figure 4D). PERMANOVAs of Bray–Curtis distances indicated that 29.0% of the variation was explained by colony, 6.2% by pesticide treatment, and 9.3% by their interaction. Similarly, Jaccard distances indicated that 19.7% of the variation is explained by colony, 5.3% by treatment, and 8.7% by their interaction, and Unifrac distances gave that 38.8% of the variation is explained by colony, 8.6% by treatment, and 8.6% by their interaction (Supplementary Figure 6). For all diversity metrics, compared to controls, oxalic acid harbored the most distinct microbiome (Bray–Curtis: *F*_1_,_66_ = 8.444; *p* < 0.0001, Jaccard: *F*_1_,_66_ = 5.557; *p* < 0.0001, Unifrac: *F*_1_,_66_ = 17.6; *p* < 0.0001), followed by acetamiprid (Bray–Curtis: *F*_1_,_69_ = 2.641; *p* = 0.0305, Jaccard: *F*_1_,_69_ = 2.151; *p* = 0.0278, Unifrac: *F*_1_,_69_ = 0.769; *p* = 0.473) and thiacloprid (Bray–Curtis: *F*_1_,_70_ = 2.065; *p* = 0.0557, Jaccard: *F*_1_,_70_ = 1.844; *p* = 0.0370, Unifrac: *F*_1_, _70_ = 0.348; *p* = 0.827).

### Treatment Affects Microbiome Composition, Particularly After Oxalic Acid Exposure

**FIGURE 5:**
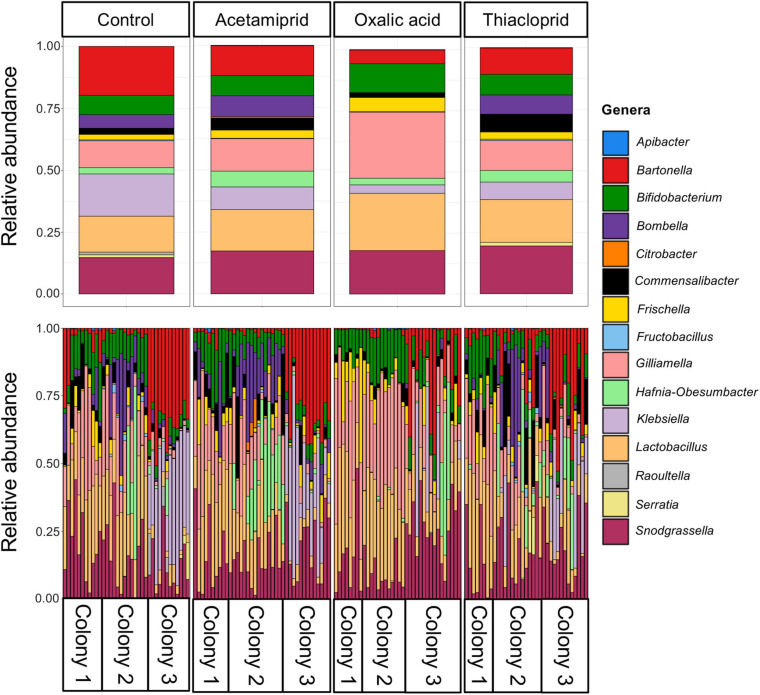
Relative abundances of the 15 most abundant bacterial genera across the full dataset. The top panel gives relative abundances by treatment, while the bottom panel gives the relative abundances per honeybee gut, illustrating the abundant variation across samples within and between colonies.

Across the full dataset, the most abundant genera were *Snodgrassella*, *Lactobacillus*, *Gilliamella*, *Commensalibacter*, *Frischella*, *Bombella*, *Bifidobacterium*, and *Bartonella* (Figure 5 and Supplementary Table 9), accounting for 87.2% of all clean sequence reads, and consistent with previous findings (Engel et al., 2012; Bonilla-Rosso and Engel, 2018; Ellegaard and Engel, 2019). There was, however, extensive variation both between colonies and, to a lesser extent, between bees from the same colony (Figure 5). *Hafnia–Obesumbacterium* and *Klebsiella*, two environmental bacteria that are opportunistically associated with honeybees, were abundant in some samples, with an overall average abundance of 3.9 and 9.7%, respectively. However, the abundance of these opportunistic bacteria was not associated with pesticide treatment (Figure 5 and Supplementary Table 9). Their elevated levels, including in controls, may have been due to the presence of dead bees within the boxes, which could have acted as reservoirs for the transfer of opportunistic and pathogenic bacteria to live bees. Future work should thus consider removal of dead bees to prevent effects on microbiomes of focal individuals.

**FIGURE 6:**
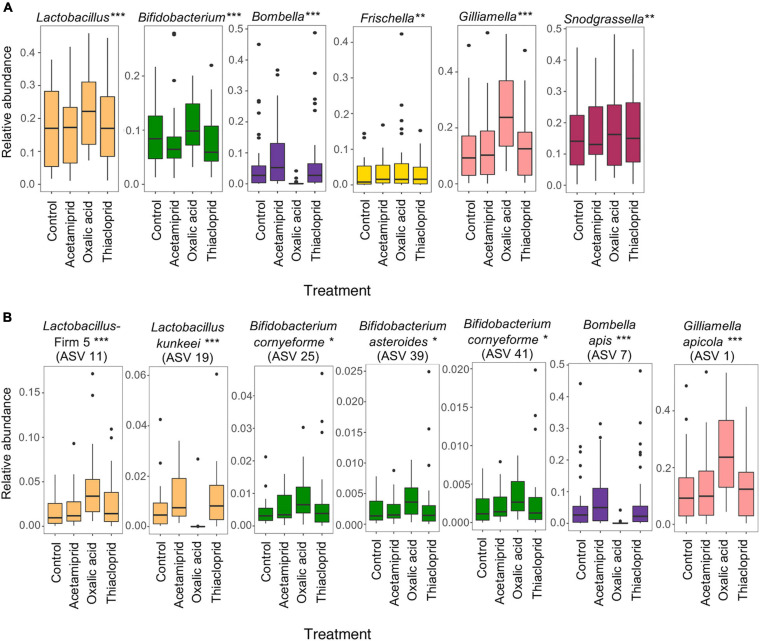
Oxalic acid treatment changes microbial relative abundance *in vivo*. **(A)** Relative abundance of core microbes at the genus level between controls and different pesticide treatments. **(B)** Relative abundance of differentially abundant ASVs between control and pesticide treatments. Asterisks indicate significant changes between control and oxalic acid treatments assessed in two independent ALDEx2 bivariate models with pesticide treatment and colony as fixed effects (*p < 0.05, **p < 0.01, ***p < 0.001). Acetamiprid and thiacloprid are not significantly different from controls. Boxplots represent the first and third quartiles of the relative abundance, the horizontal line represents the median, whiskers extend 1.5 interquartile ranges and dots represent outliers.

To better understand the community changes underlying differences in alpha and beta diversity, we assessed the differential abundance of genera (Figure 6A) and ASVs (Figure 6B) between respective treatments and controls. We did not find any differentially abundant genera nor ASVs between controls and acetamiprid or thiacloprid treatments. However, six genera and seven ASVs differed in relative abundance between controls and oxalic acid treated bees. At the genus level, the relative abundance of *Bombella* was significantly reduced after oxalicacid treatment (ALDEx2 test: *t* = −6.054, *p* < 0.0001), while *Gilliamella* (*t* = 8.989, *p* < 0.0001), *Frischella* (*t* = 4.030, *p* = 0.0023), *Lactobacillus* (*t* = 7.986, *p* < 0.0001), *Bifidobacterium* (*t* = 6.912, *p* < 0.0001), and *Snodgrassella* (*t* = 3.942, *p* = 0.0033) all increased in relative abundance in response to the oxalic acid treatment (Figure 6A). At the ASV level, *Bombella intestini* (*t* = −8.326, *p* < 0.0001) and *L. kunkeei* (*t* = −10.56, *p* < 0.0001; Supplementary Figure 7) were negatively affected by oxalic acid. Conversely, a *Lactobacillus* Firm-5 ASV (*t* = 7.505, *p* < 0.0001), the most abundant *Gilliamella* ASV (*t* = 7.379, *p* < 0.0001), and three *Bifidobacterium* ASVs (*t* = 4.543, *p* = 0.0157; *t* = 4.210, *p* = 0.0251; and *t* = 4.067, *p* = 0.0348) were all relatively more abundant in oxalic acid treated bees than in controls (Figure 6B). These changes in relative abundance of genera and ASVs reflect altered community composition. However, differences in the absolute abundances would require quantification of the 16S rRNA gene to estimate bacterial load, and should be considered in future studies.

### Pesticide Treatment Affects Network Relationships Between Honeybee Gut Microbes

**FIGURE 7:**
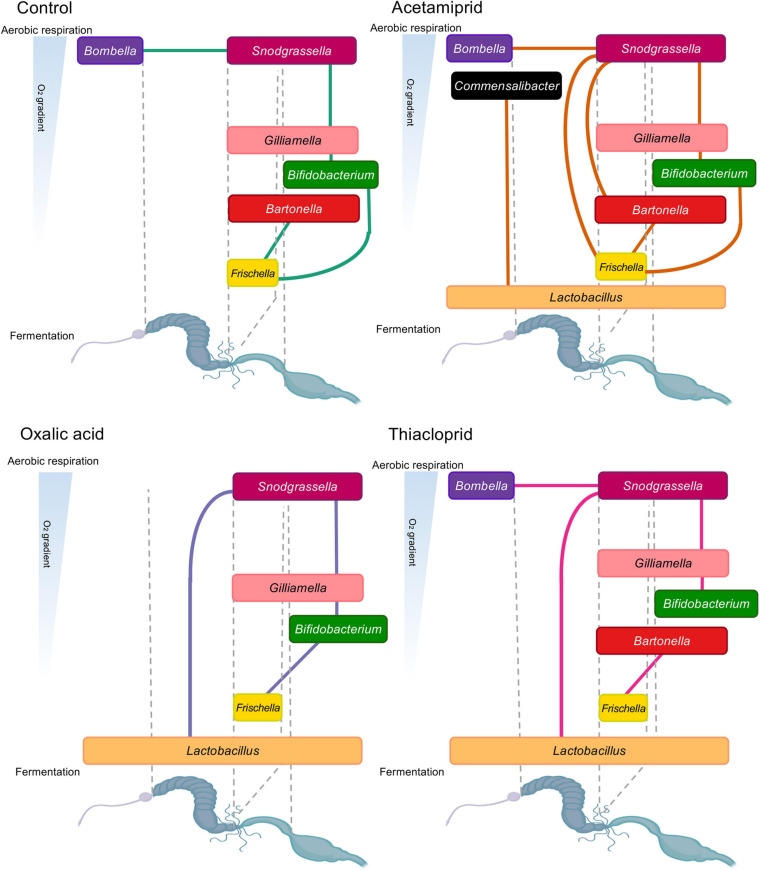
Positive relationships in honeybee gut bacterial genera by experimental treatment. Lines between bacterial genera indicate positive relationships from individual network analyses. The genera are represented based on their oxygen niche (*y*-axis) and their position in the gut (*x*-axis), with the width of text boxes indicative of their gut placement. The oxygen niche and position in the gut is based on current literature (Anderson et al., 2013; Kwong and Moran, 2016; Butler et al., 2013; Kešnerová et al., 2017; Bonilla-Rosso and Engel, 2018).

To elucidate potential syntrophic relationships among the identified genera in our dataset, and to assess whether these were affected by pesticide treatment, we performed network analyses for controls and each treatment, pooling data from all colonies (Figure 7). Overall, we observed flexibility in ecological networks of pesticide-treated microbiomes, but some positive associations were shared with controls (Figure 7). All positive interactions in controls were present in the acetamiprid treatment (Figure 7), but here we also saw associations between *Commensalibacter* and *Lactobacillus*, and between *Snodgrassella* and *Frischella* and *Bartonella*. In oxalic acid-treated honeybees (Figure 7), the association between *Bombella* and *Snodgrassella* present in controls disappeared, likely due to *Bombella* sensitivity to this pesticide. Additionally, *Snodgrassella* was linked to *Lactobacillus*, which potentially fills *Bombella*’s niche of aerobic respiration. Under thiacloprid treatment (Figure 7), it is noteworthy that we could not replicate the *Bifidobacterium–Frischella* association, while a *Lactobacillus*–*Snodgrassella* association emerged. Finally, associations between *Lactobacillus* and other core members were surprisingly lacking in the controls, despite ample evidence of syntrophic relationships (Kwong and Moran, 2016). The lack of association may be due to the variable niches and interbacterial interactions of *L.* Firm 4, *L.* Firm 5, and *L. kunkeei* (Bonilla-Rosso and Engel, 2018) that lead to variable levels of *Lactobacillus* and inconsistent positive correlation with other bacteria. Nevertheless, overall changes in interbacterial networks point toward the presence of either a flexible or a fragile ecological network in the honeybee gut microbiome.

## Discussion

We examined the effect that three commonly used pesticides have on microbial symbionts of honeybees and found evidence for both *in vitro* and *in vivo* effects of oxalic acid exposure, but no effects of acetamiprid or thiacloprid exposure in the *in vitro* assay and far less marked effects *in vivo*. It is apparent that *in vitro* testing alone is insufficient to deduce effects on honeybee gut microbiomes and ultimately honeybee health. Although we were limited from quantifying the amount of liquid that bees consumed within sub-colonies late in the experiment, when fewer bees were present, all sub-colonies appeared to consume sugar water, indicating exposure. Oxalic acid was the only pesticide for which we observed reduced number of days with consumption, consistent with previous suggestions that palatability may cause oxalic acid avoidance (Nanetti et al., 2015). Its consumption is nevertheless corroborated by the observed effects on gut microbial communities, with reduced microbial richness, reduced abundance of key microbiome taxa, and altered interbacterial relationships. We did not see consistently higher mortality induced by the presence of either of the three compounds compared to controls, so longer-term exposures may be needed to elucidate any such effects.

It should be noted that we observed very high mortality across all experimental groups, suggesting that caution should be taken when interpreting beyond laboratory experimental group comparisons. This high mortality was likely due to a combination of factors. The low alpha diversity in field control samples (see Supplementary Text) may suggest that the colonies were not in optimal health condition at the time of sampling (September). However, the alpha diversity and microbiome composition is known to change as the bees transition to the winter season, but the impact of this change on bee health remains unclear (Ludvigsen et al., 2015; Bleau et al., 2020; Kešnerová et al., 2020). Furthermore, although we conceivably avoided foragers (older bees) by sampling from the bottom of the hive frames, we did not strictly age control and may thus have included older bees with a higher risk of dying during the experiment. However, two other factors have conceivably played a more important role in governing mortality. First, honeybees are sensitive to stress (Klein et al., 2017), and transport and the use of CO_2_ to anesthetize bees may have negative impacts. Secondly, to minimize disturbance and the risk of escapees we did not remove dead bees during the experiment, and it is likely that this has increased risks of cross infections with opportunistic pathogens from dead to live bees (see results on the elevated levels of opportunistic pathogens in control bees). Although such high mortality is not optimal, the consistent patterns observed in the microbiome analyses, and the impacts we see on specific core gut microbes, warrant meaningful comparisons and reflect biologically relevant effects.

### Do *in vitro* Observations Predict *in vivo* Effects?

We did not observe *in vitro* inhibition by thiacloprid or acetamiprid of any of the tested bacteria, and the communities treated with these pesticides *in vivo* were only slightly altered compared to controls. Oxalic acid inhibition *in vitro* was consistent with greater alteration of the gut microbiomes *in vivo*, but the bacteria affected *in vivo* differed from those affected *in vitro*. This, and its comparably wider use as an approved pesticide, lead us to focus our discussion on potential impacts of oxalic acid exposure.

The outcome of pesticide treatment on gut bacterial abundances *in vivo* should be the combined effect of bacterial sensitivity, direct exposure and interbacterial dependencies. Bacteria that are sensitive *in vitro* to 0.95–9.5 mg/ml, conceivably comparable to potential exposures in the field, appear to increase in relative abundance *in vivo* after treatment with oxalic acid compared to controls. This is likely due to exposure differences *in vitro* and within bee guts, and illustrates the limitations that plating experiments have for evaluating impacts of anthropogenic compounds on host-associated microbes. Exposure is inevitable *in vitro*, but this is not necessarily so *in vivo*. For example, biofilm formation helps protect from environmental changes, including chemical or antibiotic exposure (Singh et al., 2017). Sensitivity to oxalic acid in the *in vitro* experiment would predict effects on e.g., *G. apicola, F. perrara, B. asteroides*, and *S. alvi in vivo.* However, within bees, *G. apicola* interacts in a biofilm with *S. alvi* and engages in cross-feeding interactions (Engel et al., 2012; Kešnerová et al., 2017). Such close syntrophic interactions within multispecies biofilms are likely to reduce oxalic acid exposure or buffer its effects.

Another potential explanation for the reduced impact on most core bacteria is their location in the intestinal tract. Apart from *L. kunkeei* and *B. apis*, the two microbes depleted by oxalic acid *in vivo*, all the tested bacteria reside in the hindgut (Anderson et al., 2013; Moran, 2015; Kwong and Moran, 2016; Powell et al., 2018). The hindgut receives pre-digested material from the midgut, and oxalic acid may thus at least partly have been digested before reaching the hindgut (Engel and Moran, 2013). A combination of decreasing oxalic acid concentrations along the bee gut and biofilm formation in the hindgut seems plausible to explain the effects on the honeybee gut microbes, suggesting that the most profound implications of exposure for colony health is conceivably effects on crop bacteria. In this context, we should note that increases in relative abundances are likely driven mainly by the loss of other bacteria, increasing the relative abundance of taxa in the microbiome that are unaffected by pesticide treatment. In order to determine if treatment affects the absolute abundance of bacteria, and to explore potential relationships between bacterial abundances and alpha diversity, bacterial load would need to be quantified.

### Implications of Oxalic Acid Bacterial Inhibition on Colony Dynamics

*In vitro* inhibition of core honeybee microbes and *in vivo* depletion of crop members are potentially concerning for honeybee colony health. Bacteria move within colonies by trophallaxis between bees (Martinson et al., 2012), which includes the exchange of crop contents, via bees picking up bacteria from hive material (Kwong and Moran, 2016), and through consumption of hindgut exudates (Powell et al., 2014). Oxalic acid treatment by trickling depends on the redistribution of the pesticide by the bees themselves (Schneider et al., 2012; Rademacher et al., 2017). While bacteria in most gut compartments may be somewhat protected from oxalic acid, we do observe a significant overall negative effect on microbiome richness, estimated as the number of ASVs bee guts contain. The regeneration of the microbiome after local extinctions in individual bees, or during inoculation of young bees, also depends on bacteria moving from hive to bee and between bees, implying that colony-level impacts from treatment with oxalic acid are likely.

Other studies have found oxalic acid inhibition *in vitro* of both honeybee microbes (Diaz et al., 2019) and plant pathogens (Kwak et al., 2016), and oxalic acid is present in honey and has been suggested to be responsible for its antimicrobial properties (Bogdanov et al., 2002; Nozal et al., 2003). While most strains appear resistant to oxalic acid in low concentrations, oxalic acid can persist in colonies for multiple weeks (Rademacher et al., 2017). If accumulation occurs after multiple oxalic acid treatments, the pesticide may have a stronger effect on honeybee microbes. Regular oxalic acid application to colonies may also select for resistance in opportunistic bacteria, *Varroa*, and honeybee mutualists and commensals. This may impair the function constitutive levels of oxalic acid have in honeybee colonies, ultimately requiring treatment with higher concentrations with impacts on colony health and production (Adjlane et al., 2016). As *Varroa* has a larger impact on honeybee health on the short-term than oxalic acid treatment, we advise exploring genetic resistance to *Varroa* in honeybees, as it may be a better long-term solution against infection than the application of oxalic acid (Conlon et al., 2019).

### Implications of Pesticide Treatment on Honeybee Health

Oxalic acid impact on the honeybee gut microbiome could be direct or indirect. Indirect effects could be mediated by necrotic cell death in the midgut (Gregorc and Smodiš Škerl, 2007; Papežíková et al., 2017), lesions (Martín-Hernández et al., 2007), or pH changes (Rademacher et al., 2017). Since the affected microbes are present in the crop, rather than the midgut or hindgut, and the temporal scope of our experiment is restrictive, we can with some confidence rule out necrotic cell death and lesions for our experiment. This leaves pH changes as the most likely effector, which could have secondary effects on nutrient digestion and absorption.

Our network analyses replicate previously published data on interbacterial relationships (Kwong and Moran, 2016; Kešnerová et al., 2017; Ellegaard and Engel, 2019). Encouragingly, they revealed that interbacterial relationships are potentially flexible, at least on the short-term, with the caveat that our relationships are inferred by correlation, and hence may not reflect actual syntrophic or other types of relationships. Another interpretation is they are relatively fragile to environmental stressors. Crop bacteria are responsible for a portion of aerobic metabolism in the bee gut, therefore creating an anaerobic environment in lower portions of the gut. However, even after severe depletion of crop bacteria with oxalic acid, it seems that interbacterial relationships reassemble in such a way that *Snodgrassella* is now responsible for oxygen metabolism and depletion, leading to an association between *Snodgrassella* and fermentative Lactobacilli (Kešnerová et al., 2017). *Snodgrassella*’s association with the facultative aerobe *Gilliamella* remains intact under this pesticide treatment, as do *Gilliamella–Bifidobacterium* and *Bifidobacterium–Frischella* associations, all of which exist in the lower portions of the honeybee gut. The main impacts on interbacterial relationships are therefore captured by the disappearance of *Bombella* and *L. kunkeei*, which affect other relationships, such as those involving *Bartonella*.

*Lactobacillus kunkeei* is one of the most abundant bacteria in the honeybee crop, and is also found in nectar, pollen, and honey (Anderson et al., 2013; Corby-Harris et al., 2014a; Bonilla-Rosso and Engel, 2018; Kwong and Moran, 2016). *In vitro*, we see that this species is relatively more resistant, potentially due to exposure of oxalic acid in honey (Bogdanov et al., 2002; Nozal et al., 2003). *Lactobacillus* presence increases brood and honey production (Alberoni et al., 2017), improves the honeybee immune response (Maruščáková et al., 2020), protects against foulbrood diseases (Vásquez et al., 2012) and some insect-associated strains can metabolize insecticides (De Almeida et al., 2017; Daisley et al., 2018). The strain-level turnover between *Lactobacillus* strains we see in our experiment may have implications for colony health, as it is an important factor for honeybee microbiome function (Ellegaard et al., 2020). For example, *L. kunkeei* presence decreases larval mortality during *Paenibacillus larvae* infection and decreases *Nosema* infection prevalence in adults (Arredondo et al., 2018). Encouragingly, *L. kunkeei* can regrow surprisingly quickly after oxalic acid has been removed from the colony environment (Supplementary Figure 6). The other affected genus in our study, *Bombella*, has very specific niches within bees, including in nurse hypopharyngeal glands from which larvae are feed, the crop of nurse bees, and in royal jelly. Its location in the crop of nurses and in royal jelly implies potentially relevant roles in larvae and queen development (Corby-Harris et al., 2014b; Tarpy et al., 2015). Consistent with this assertion, it comprises a large fraction of the queen gut microbiome, possibly playing a role in queen nutrition and in modulating queen fertility, fecundity, and longevity (Anderson et al., 2018). This may involve protection from pathogens, as indicated by *Bombella apis* supplementation in colonies significantly reducing *Nosema* prevalence (Corby-Harris et al., 2016). Colony-level effects of oxalic acid treatment on *Bombella* must thus be assessed, as our results could have implications for honeybee immunity, longevity and fecundity.

Acetamiprid and thiacloprid treatment did not inhibit bacterial growth *in vitro* or impact the richness of honeybee gut microbiota compared to controls *in vivo*. Acetamiprid and thiacloprid treatments generate little change in the gut communities of treated bees, maintaining richness levels and core member relative diversity observed in the lab controls. Acetamiprid has not been investigated for its potential detrimental impact on honeybee gut microbiome, but chronic exposure of its fellow neonicotinoids, like thiamethoxam or imidacloprid, greatly alter intestinal communities of bees (Rouzé et al., 2019). Our *in vivo* findings contrast recent work by Liu et al. (2020) who described gut bacterial dysbiosis in honeybees exposed to thiacloprid in a dose dependent manner after one week of exposure; however, our experiment tested lower concentrations (0.05 mg/L), so this may explain the lower effect of thiacloprid. The bacterial communities recovered by day thirteen in Liu et al. (2020), likely due to anal trophallaxis between colony members compensating for the loss of honeybee gut members (Kwong and Moran, 2016) and could also be buffering the effect of thiacloprid in our experiment.

## Data Availability Statement

The 16S rRNA datasets generated in this study can be found in the SRA archive in GenBank under the BioProject PRJNA732842.

## Author Contributions

AC-M, AJ, and MP conceived and designed the experiment. AC-M and PK collected the bees. AC-M performed the laboratory work with assistance from VS. JR-H, AC-M, and VS analyzed the community data while AC-M and VS analyzed *in vitro*, mortality, and consumption data. AC-M created the first draft of the manuscript and figures with VS, MP, JR-H, and AJ further editing and providing feedback. All authors agreed on the final manuscript.

## Conflict of Interest

The authors declare that the research was conducted in the absence of any commercial or financial relationships that could be construed as a potential conflict of interest.

## Publisher’s Note

All claims expressed in this article are solely those of the authors and do not necessarily represent those of their affiliated organizations, or those of the publisher, the editors and the reviewers. Any product that may be evaluated in this article, or claim that may be made by its manufacturer, is not guaranteed or endorsed by the publisher.

## Footnotes

1
https://microbe.med.umich.edu/welcome-university-michigan%E2%80%99s-microbiome-core

## References

[B1] Adjlane N. Tarek E. O. Haddad N. (2016). Evaluation of oxalic acid treatments against the mite *Varroa destructor* and secondary effects on honeybees *Apis mellifera*. *J. Arthropod Borne Dis.* 10 501–509. 28032102 PMC5186740

[B2] Alberoni D. Baffoni L. Gaggìa F. Ryan P. M. Murphy K. Ross P. R. (2017). Impact of beneficial bacteria supplementation on the gut microbiota, colony development and productivity of *Apis mellifera* L. *Benef. Microbes* 9 269–278. 10.3920/BM2017.0061 29380644

[B3] Amulen D. R. Spanoghe P. Houbraken M. Tamale A. De Graaf D. C. Cross P. (2017). Environmental contaminants of honeybee products in Uganda detected using LC-MS/MS and GC-ECD. *PLoS One* 12:e0178546. 10.1371/journal.pone.0178546 28570581 PMC5453540

[B4] Anderson K. E. Ricigliano V. A. Mott B. M. Copeland D. C. Floyd A. S. Maes P. (2018). The queen’s gut refines with age: longevity phenotypes in a social insect model. *Microbiome* 6:108. 10.1186/s40168-018-0489-1 29914555 PMC6006926

[B5] Anderson K. E. Sheehan T. H. Mott B. M. Maes P. Snyder L. Schwan M. R. (2013). Microbial ecology of the hive and pollination landscape: bacterial associates from floral nectar, the alimentary tract and stored food of honey bees (*Apis mellifera*). *PLoS One* 8:e83125. 10.1371/journal.pone.0083125 24358254 PMC3866269

[B6] Arredondo D. Castelli L. Porrini M. P. Garrido P. M. Eguaras M. J. Zunino P. (2018). *Lactobacillus kunkeei* strains decreased the infection by honey bee pathogens *Paenibacillus larvae* and *Nosema ceranae*. *Benef. Microb*. 9, 279–290. 10.3920/BM2017.0075 29264966

[B7] Balouiri M. Sadiki M. Ibnsouda S. K. (2016). Methods for *in vitro* evaluating antimicrobial activity: a review. *J. Pharm. Anal.* 6 71–79. 10.1016/j.jpha.2015.11.005 29403965 PMC5762448

[B8] Begna T. Jung C. (2021). Effects of sequential exposures of sub-lethal doses of amitraz and thiacloprid on learning and memory of honey bee foragers, *Apis mellifera*. *J. Asia Pac. Entomol.* 24 77–83. 10.1016/j.aspen.2021.03.012

[B9] Binetruy F. Dupraz M. Buysse M. Duron O. (2019). Surface sterilization methods impact measures of internal microbial diversity in ticks. *Parasit. Vectors* 12 1–10. 10.1186/s13071-019-3517-5 31138324 PMC6537145

[B10] Bleau N. Bouslama S. Giovenazzo P. Derome N. (2020). Dynamics of the honeybee (*Apis mellifera*) gut microbiota throughout the overwintering period in Canada. *Microorganisms* 8 1–11. 10.3390/microorganisms8081146 32751209 PMC7464175

[B11] Blot N. Veillat L. Rouzé R. Delatte H. (2019). Glyphosate, but not its metabolite AMPA, alters the honeybee gut microbiota. *PLoS One* 14:e0215466. 10.1371/journal.pone.0215466 30990837 PMC6467416

[B12] Bogdanov S. Charrière J. D. Imdorf A. Kilchenmann V. Fluri P. (2002). Determination of residues in honey after treatments with formic and oxalic acid under field conditions. *Apidologie* 33 399–409. 10.1051/apido:2002029

[B13] Boncristiani H. Underwood R. Schwarz R. Evans J. D. Pettis J. vanEngelsdorp D. (2012). Direct effect of acaricides on pathogen loads and gene expression levels in honey bees *Apis mellifera*. *J. Insect Physiol.* 58 613–620. 10.1016/j.jinsphys.2011.12.011 22212860

[B14] Bonilla-Rosso G. Engel P. (2018). Functional roles and metabolic niches in the honey bee gut microbiota. *Curr. Opin. Microbiol.* 43 69–76. 10.1016/j.mib.2017.12.009 29309997

[B15] Brandt A. Grikscheit K. Siede R. Grosse R. Meixner M. D. Büchler R. (2017). Immunosuppression in Honeybee Queens by the Neonicotinoids Thiacloprid and Clothianidin. *Sci. Rep.* 7:4673. 10.1038/s41598-017-04734-1 28680118 PMC5498664

[B16] Breeze T. D. Vaissière B. E. Bommarco R. Petanidou T. Seraphides N. Kozák L. (2014). Agricultural policies exacerbate honeybee pollination service supply-demand mismatches across Europe. *PLoS One* 9:e82996. 10.1371/journal.pone.0082996 24421873 PMC3885438

[B17] Breusch T. S. Pagan A. R. (1979). A Simple Test for Heteroscedasticity and Random Coefficient Variation. *Econometrica* 47 1287–1294. 10.2307/1911963

[B18] Brodschneider R. Brus J. Danihlík J. (2019). Comparison of apiculture and winter mortality of honey bee colonies (*Apis mellifera*) in Austria and Czechia. *Agric. Ecosyst. Environ.* 274 24–32. 10.1016/j.agee.2019.01.002

[B19] Brosi B. J. Delaplane K. S. Boots M. De Roode J. C. (2017). Ecological and evolutionary approaches to managing honeybee disease. *Nat. Ecol. Evol.* 1 1250–1262. 10.1038/s41559-017-0246-z 29046562 PMC5749923

[B20] Butler È Alsterfjord M. Olofsson T. C. Karlsson C. Malmström J. Vásquez A. (2013). Proteins of novel lactic acid bacteria from *Apis mellifera mellifera*: an insight into the production of known extra-cellular proteins during microbial stress. *BMC Microbiol*. 13:235. 10.1186/1471-2180-13-235 24148670 PMC4015849

[B21] Callahan B. McMurdie P. Rosen M. Han A. W. Johnson A. J. Holmes S. (2016). P. DADA2: high-resolution sample inference from Illumina amplicon data. *Nat Methods* 13 581–583. 10.1038/nmeth.3869 27214047 PMC4927377

[B22] Carreck N. L. Andree M. Brent C. S. Cox-Foster D. Dade H. A. Ellis J. D. (2013). Standard methods for *Apis mellifera* anatomy and dissection. *J. Apic. Res.* 52:4. 10.3896/IBRA.1.52.4.03

[B23] Casida J. E. Durkin K. A. (2013). Neuroactive insecticides: targets, selectivity, resistance, and secondary effects. *Ann. Rev. Entomol.* 58 99–117. 10.1146/annurev-ento-120811-153645 23317040

[B24] Charreton M. Decourtye A. Henry M. Rodet G. Sandoz J. C. Charnet P. (2015). A locomotor deficit induced by sublethal doses of pyrethroid and neonicotinoid insecticides in the honeybee *Apis mellifera*. *PLoS One* 10:e0144879. 10.1371/journal.pone.0144879 26659095 PMC4682844

[B25] Charrière J. D. Imdorf A. (2002). Oxalic acid treatment by trickling against *Varroa destructor*: recommendations for use in central Europe and under temperate climate conditions. *Bee World* 83 51–60. 10.1080/0005772X.2002.11099541

[B26] Conlon B. H. Aurori A. Giurgiu A. I. Kefuss J. Dezmirean D. S. Moritz R. F. A. (2019). A gene for resistance to the *Varroa* mite (*Acari*) in honey bee (*Apis mellifera*) pupae. *Mol. Ecol.* 28 2958–2966. 10.1111/mec.15080 30916410

[B27] Corby-Harris V. Maes P. Anderson K. E. (2014a). The bacterial communities associated with honey bee (*Apis mellifera*) foragers. *PLoS One* 9:e95056. 10.1371/journal.pone.0095056 24740297 PMC3989306

[B28] Corby-Harris V. Snyder L. Meador C. A. D. Naldo R. Mott B. Anderson K. E. (2016). *Parasaccharibacter apium*, gen. Nov., sp. Nov., Improves Honey Bee (*Hymenoptera*: *Apidae*) resistance to Nosema. *J. Econ. Entomol.* 109 537–543. 10.1093/jee/tow012 26875068

[B29] Corby-Harris V. Snyder L. A. Schwan M. R. Maes P. Mcfrederick Q. S. Anderson E. (2014b). Origin and Effect of Alpha 22 *Acetobacteraceae* in Honey Bee Larvae. *Appl. Environ. Microbiol*. 80 7460–7472. 10.1128/AEM.02043-14 25239902 PMC4249245

[B30] Dacher M. Lagarrigue A. Gauthier M. (2005). Antennal tactile learning in the honeybee: effect of nicotinic antagonists on memory dynamics. *Neuroscience* 130 37–50. 10.1016/j.neuroscience.2004.09.006 15561423

[B31] Daisley B. A. Trinder M. McDowell T. W. Collins S. L. Sumarah M. W. Reid G. (2018). Microbiota-mediated modulation of organophosphate insecticide toxicity by species-dependent interactions with lactobacilli in a *Drosophila melanogaster* insect model. *Appl. Environ. Microbiol.* 84 1–13. 10.1128/AEM.02820-17 29475860 PMC5930343

[B32] De Almeida L. G. De Moraes L. A. B. Trigo J. R. Omoto C. Cônsoli F. L. (2017). The gut microbiota of insecticide-resistant insects houses insecticide-degrading bacteria: a potential source for biotechnological exploitation. *PLoS One* 12:e0174754. 10.1371/journal.pone.0174754 28358907 PMC5373613

[B33] Decourtye A. Armengaud C. Renou M. Devillers J. Cluzeau S. Gauthier M. (2004a). Imidacloprid impairs memory and brain metabolism in the honeybee (*Apis mellifera L*.). *Pestic. Biochem. Physiol.* 78 83–92. 10.1016/j.pestbp.2003.10.001

[B34] Decourtye A. Devillers J. Cluzeau S. Charreton M. Pham-Delègue M. H. (2004b). Effects of imidacloprid and deltamethrin on associative learning in honeybees under semi-field and laboratory conditions. *Ecotoxicol. Environ. Saf.* 57 410–419. 10.1016/j.ecoenv.2003.08.001 15041263

[B35] Dewar A. M. (2017). The adverse impact of the neonicotinoid seed treatment ban on crop protection in oilseed rape in the United Kingdom. *Pest Manag. Sci.* 73 1305–1309. 10.1002/ps.4511 28019077

[B36] Diaz T. del-Val E. Ayala R. Larsen J. (2019). Alterations in honey bee gut microorganisms caused by *Nosema* spp. and pest control methods. *Pest Manag. Sci.* 75 835–843. 10.1002/ps.5188 30151856

[B37] El Hassani A. K. Dacher M. Gary V. Lambin M. Gauthier M. Armengaud C. (2008). Effects of sublethal doses of acetamiprid and thiamethoxam on the behavior of the honeybee (*Apis mellifera*). *Arch. Environ. Contam. Toxicol.* 54 653–661. 10.1007/s00244-007-9071-8 18026773

[B38] Ellegaard K. M. Engel P. (2019). Genomic diversity landscape of the honey bee gut microbiota. *Nat. Commun.* 10:446. 10.1038/s41467-019-08303-0 30683856 PMC6347622

[B39] Ellegaard K. M. Suenami S. Miyazaki R. Engel P. (2020). Vast Differences in Strain-Level Diversity in the Gut Microbiota of Two Closely Related Honey Bee Species. *Curr. Biol.* 30 2520–2531.e7. 10.1016/j.cub.2020.04.070 32531278 PMC7342003

[B40] Engel P. Moran N. A. (2013). The gut microbiota of insects - diversity in structure and function. *FEMS Microbiol. Rev.* 37 699–735. 10.1111/1574-6976.12025 23692388

[B41] Engel P. Martinson V. G. Moran N. A. (2012). Functional diversity within the simple gut microbiota of the honey bee. *Proc. Natl. Acad. Sci. U.S.A.* 109, 11002–11007. 10.1073/pnas.1202970109 22711827 PMC3390884

[B42] Engel P. James R. R. Koga R. Kwong W. K. McFrederick Q. S. Moran N. A. (2013). Standard methods for research on *Apis mellifera* gut symbionts. *J. Apic. Res.* 52 1–24. 10.3896/IBRA.1.52.4.07

[B43] Espregueira Themudo G. Rey-Iglesia A. Robles Tascón L. Bruun Jensen A. da Fonseca R. R. Campos P. F. (2020). Declining genetic diversity of European honeybees along the twentieth century. *Sci. Rep.* 10:10520. 10.1038/s41598-020-67370-2 32601293 PMC7324561

[B44] European commission (2013). *European commission.* Available online at: https://ec.europa.eu/food/plant/pesticides/approval_active_substances/approval_renewal/neonicotinoids_en (accessed May 23, 2021).

[B45] European Commission (2020). *European Commission*. Available online at: https://ec.europa.eu/newsroom/sante/items/667420/en (accessed August 10, 2021).

[B46] European Food Safety Authority (EFSA) (2020). *European Food Safety Authority.* Available online at: https://www.efsa.europa.eu/en/news/pesticides-efsa-examine-emergency-use-neonicotinoids (accessed May 23, 2021).

[B47] Evans J. D. Chen Y. P. di Prisco G. Pettis J. Williams V. (2009). Bee cups: single-use cages for honeybee experiments. *J. Apic. Res.* 48 300–302. 10.1080/00218839.2009.11101548

[B48] Fernandes A. D. Macklaim J. M. Linn T. G. Reid G. Gloor G. B. (2013). ANOVA-Like Differential Expression (ALDEx) Analysis for Mixed Population RNA-Seq. *PLoS One* 8:e67019. 10.1371/journal.pone.0067019 23843979 PMC3699591

[B49] Gill R. J. Ramos-Rodríguez O. Raine N. E. (2012). Combined pesticide exposure severely affects individual- and colony-level traits in bees. *Nature* 490 105–108. 10.1038/nature11585 23086150 PMC3495159

[B50] Goulson D. Nicholls E. Botías C. Rotheray E. L. (2015). Bee declines driven by combined Stress from parasites, pesticides, and lack of flowers. *Science* 347:1255957. 10.1126/science.1255957 25721506

[B51] Gregorc A. Smodiš Škerl M. I. (2007). Toxicological and immunohistochemical testing of honeybees after oxalic acid and rotenone treatments. *Apidologie* 38 296–305. 10.1051/apido:2007014

[B52] Hristov P. Neov B. Shumkova R. Palova N. (2020). Significance of apoidea as main pollinators. ecological and economic impact and implications for human nutrition. *Diversity* 12:280. 10.3390/d12070280

[B53] Huang S. K. Csaki T. Doublet V. Dussaubat C. Evans J. D. Gajda A. M. (2014). Evaluation of cage designs and feeding regimes for honeybee (Hymenoptera: *Apidae*) laboratory experiments. *J. Econ. Entomol.* 107 54–62. 10.1603/EC13213 24665684

[B54] Hung K. L. J. Kingston J. M. Albrecht M. Holway D. A. Kohn J. R. (2018). The worldwide importance of honeybees as pollinators in natural habitats. *Proc. R. Soc. B Biol. Sci.* 285:20172140. 10.1098/rspb.2017.2140 29321298 PMC5784195

[B55] Iwasa T. Motoyama N. Ambrose J. T. Roe R. M. (2004). Mechanism for the differential toxicity of neonicotinoid insecticides in the honeybee, *Apis mellifera*. *Crop Prot.* 23 371–378. 10.1016/j.cropro.2003.08.018

[B56] Johnson R. M. (2015). Honeybee toxicology. *Annu. Rev. Entomol.* 60 415–434. 10.1146/annurev-ento-011613-162005 25341092

[B57] Jones J. C. Fruciano C. Marchant J. Hildebrand F. Forslund S. Bork P. (2018). The gut microbiome is associated with behavioural task in honey bees. *Insectes Soc.* 65 419–429. 10.1007/s00040-018-0624-9 30100619 PMC6061168

[B58] Kakumanu M. L. Reeves A. M. Anderson T. D. Rodrigues R. R. Williams M. A. (2016). Honeybee gut microbiome is altered by in-hive pesticide exposures. *Front. Microbiol.* 7:1255. 10.3389/fmicb.2016.01255 27579024 PMC4985556

[B59] Kešnerová L. Emery O. Troilo M. Liberti J. Erkosar B. Engel P. (2020). Gut microbiota structure differs between honeybees in winter and summer. *ISME J.* 14 801–814. 10.1038/s41396-019-0568-8 31836840 PMC7031341

[B60] Kešnerová L. Mars R. A. T. Ellegaard K. M. Troilo M. Sauer U. Engel P. (2017). Disentangling metabolic functions of bacteria in the honeybee gut. *PLoS Biol.* 15:e2003467. 10.1371/journal.pbio.2003467 29232373 PMC5726620

[B61] Kešnerová L. Moritz R. Engel P. (2016). Bartonella apis sp. nov., a honeybee gut symbiont of the class *Alphaproteobacteria*. *Int. J. Syst. Evol. Microbiol.* 66 414–421. 10.1099/ijsem.0.000736 26537852

[B62] Klein S. Cabirol A. Devaud J. M. Barron A. B. Lihoreau M. (2017). Why Bees Are So Vulnerable to Environmental Stressors. *Trends Ecol. Evol.* 32 268–278. 10.1016/j.tree.2016.12.009 28111032

[B63] Kwak A. M. Lee I. K. Lee S. Y. Yun B. S. Kang H. W. (2016). Oxalic acid from *Lentinula edodes* culture filtrate: antimicrobial activity on phytopathogenic bacteria and qualitative and quantitative analyses. *Mycobiology* 44 338–342. 10.5941/MYCO.2016.44.4.338 28154495 PMC5287170

[B64] Kwong W. K. Moran N. A. (2016). Gut Microbial Communities of Social Bees. *Nat. Rev. Microbiol.* 14 59–73. 10.1038/nrmicro.2016.43.Gut PMC5648345 27140688

[B65] Li J. H. Evans J. D. Li W. F. Zhao Y. Z. DeGrandi-Hoffman G. Huang S. K. (2017). New evidence showing that the destruction of gut bacteria by antibiotic treatment could increase the honey bee’s vulnerability to *Nosema* infection. *PLoS One* 12:e0187505. 10.1371/journal.pone.0187505 29125851 PMC5681286

[B66] Liu Y. J. Qiao N. H. Diao Q. Y. Jing Z. Vukanti R. Dai P. L. (2020). Thiacloprid exposure perturbs the gut microbiota and reduces the survival status in honeybees. *J. Hazard. Mater.* 389:121818. 10.1016/j.jhazmat.2019.121818 31818660

[B67] Ludvigsen J. Rangberg A. Avershina E. Sekelja M. Kreibich C. Amdam G. (2015). Shifts in the midgut/pyloric microbiota composition within a honey bee apiary throughout a season. *Microbes Environ.* 30 235–244. 10.1264/jsme2.ME15019 26330094 PMC4567562

[B68] Mancini F. Woodcock B. A. Isaac N. J. B. (2019). Agrochemicals in the wild: identifying links between pesticide use and declines of nontarget organisms. *Curr. Opin. Environ. Sci. Health* 11 53–58. 10.1016/j.coesh.2019.07.003

[B69] Martín-Hernández R. Higes M. Pérez J. L. Nozal M. J. Gómez L. Meana A. (2007). Short term negative effect of oxalic acid in *Apis mellifera iberiensis*. *Span. J. Agric. Res.* 5 474–480. 10.5424/sjar/2007054-270

[B70] Martinson V. G. Moy J. Moran N. A. (2012). Establishment of Characteristic Gut Bacteria during Development of the Honeybee Worker. *Appl. Environ. Microbiol.* 78 2830–2840. 10.1128/AEM.07810-11 22307297 PMC3318792

[B71] Maruščáková I. C. Schusterová P. Bielik B. Toporèák J. Bíliková K. Mudroòová D. (2020). Effect of Application of Probiotic Pollen Suspension on Immune Response and Gut Microbiota of Honey Bees (*Apis mellifera*). *Probiotics Antimicrob. Proteins* 12 929–936. 10.1007/s12602-019-09626-6 31912341

[B72] McMurdie P. J. Holmes S. (2013). Phyloseq: an R Package for Reproducible Interactive Analysis and Graphics of Microbiome Census Data. *PLoS One* 8:e61217. 10.1371/journal.pone.0061217 23630581 PMC3632530

[B73] Meeus I. Pisman M. Smagghe G. Piot N. (2018). Interaction effects of different drivers of wild bee decline and their influence on host–pathogen dynamics. *Curr. Opin. Insect Sci.* 26 136–141. 10.1016/j.cois.2018.02.007 29764653

[B74] Mills M. (2012). *Introducing Survival and Event History Analysis.* California: Sage publicactions INC.

[B75] Moosbeckhofer R. Pechhacker H. Unterweger H. Bandion F. Heinrich-Lenz A. (2003). Investigations on the oxalic acid content of honey from oxalic acid treated and untreated bee colonies. *Eur. Food Res. Technol.* 217 49–52. 10.1007/s00217-003-0698-z

[B76] Moran N. A. (2015). Genomics of the honeybee microbiome. *Curr. Opin. Insect Sci.* 10 22–28. 10.1016/j.cois.2015.04.003 26140264 PMC4484875

[B77] Moran N. A. Hansen A. K. Powell J. E. Sabree Z. L. (2012). Distinctive gut microbiota of honeybees assessed using deep sampling from individual worker bees. *PLoS One* 7:e36393. 10.1371/journal.pone.0036393 22558460 PMC3338667

[B78] Motta E. V. S. Raymann K. Moran N. A. (2018). Glyphosate perturbs the gut microbiota of honeybees. *Proc. Natl. Acad. Sci. U. S. A.* 115 10305–10310. 10.1073/pnas.1803880115 30249635 PMC6187125

[B79] Mullin C. A. Frazier M. Frazier J. L. Ashcraft S. Simonds R. vanEngelsdorp D. (2010). High Levels of Miticides and Agrochemicals in North American Apiaries: implications for Honey Bee Health. *PLoS One* 5:e9754. 10.1371/journal.pone.0009754 20333298 PMC2841636

[B80] Nanetti A. Büchler R. Charrière J. D. Fries I. Helland S. Imdorf A. (2003). Oxalic acid treatments for *Varroa* control (Review). *Apiacta* 38 81–87. 10.1016/j.rvsc.2015.08.003 26412538

[B81] Nanetti A. Rodriguez-García C. Meana A. Martín-Hernández R. Higes M. (2015). Effect of oxalic acid on Nosema ceranae infection. *Res. Vet. Sci*. 102, 167–172. 10.1016/j.rvsc.2015.08.003 26412538

[B82] Naug D. (2009). Nutritional stress due to habitat loss may explain recent honeybee colony collapses. *Biol. Conserv.* 142 2369–2372. 10.1016/j.biocon.2009.04.007

[B83] Nozal M. J. Bernal J. L. Gómez L. A. Higes M. Meana A. (2003). Determination of Oxalic acid and other organic acids in honey and in some anatomic structures of bees. *Apidologie* 34 181–188. 10.1051/apido:2003001

[B84] Oksanen J. Blanchet F. G. Friendly M. Kindt R. Legendre P. McGlinn D. (2018). *Vegan: community ecology package. R package version 2.5-2.*

[B85] Papežíková I. Palíková M. Kremserová S. Zachová A. Peterová H. Babák V. (2017). Effect of oxalic acid on the mite *Varroa destructor* and its host the honeybee *Apis mellifera*. *J. Apic. Res.* 56 400–408. 10.1080/00218839.2017.1327937

[B86] Potts S. G. Biesmeijer J. C. Kremen C. Neumann P. Schweiger O. Kunin W. E. (2010). Global pollinator declines: trends, impacts and drivers. *Trends Ecol. Evol.* 25 345–353. 10.1016/j.tree.2010.01.007 20188434

[B87] Powell J. E. Eiri D. Moran N. A. Rangel J. (2018). Modulation of the honeybee queen microbiota: effects of early social contact. *PLoS One* 13:e0200527. 10.1371/journal.pone.0200527 30001407 PMC6042773

[B88] Powell J. E. Martinson V. G. Urban-Mead K. Moran N. A. (2014). Routes of acquisition of the gut microbiota of the honeybee *Apis mellifera*. *Appl. Environ. Microbiol*. 80 7378–7387. 10.1128/AEM.01861-14 25239900 PMC4249178

[B89] Quast C. Pruesse E. Yilmaz P. Gerken J. Schweer T. Yarza P. (2013). The SILVA ribosomal RNA gene database project: improved data processing and web-based tools. *Nucleic Acids Res.* 41 590–596. 10.1093/nar/gks1219 23193283 PMC3531112

[B90] Rademacher E. Harz M. (2006). Oxalic acid for the control of varroosis in honeybee colonies - a review. *Apidologie* 37 98–120. 10.1051/apido:2005063

[B91] Rademacher E. Harz M. Schneider S. (2017). Effects of oxalic acid on *Apis mellifera* (Hymenoptera: *Apidae*). *Insects* 8:84. 10.3390/insects8030084 28783129 PMC5620704

[B92] Raymann K. Bobay L. M. Moran N. A. (2018a). Antibiotics reduce genetic diversity of core species in the honeybee gut microbiome. *Mol. Ecol.* 27 2057–2066. 10.1111/mec.14434 29164717 PMC5935549

[B93] Raymann K. Moran N. A. (2018). The role of the gut microbiome in health and disease of adult honey bee workers. *Curr. Opin. Insect Sci.* 26 97–104. 10.1016/j.cois.2018.02.012 29764668 PMC6010230

[B94] Raymann K. Motta E. V. S. Girard C. Riddington I. M. Dinser J. A. Moran N. A. (2018b). Imidacloprid decreases honey bee survival rates but does not affect the gut microbiome. *Appl. Environ. Microbiol.* 84 e00545–18. 10.1128/AEM.00545-18 29678920 PMC6007118

[B95] Raymann K. Shaffer Z. Moran N. A. (2017). Antibiotic exposure perturbs the gut microbiota and elevates mortality in honeybees. *PLoS Biol.* 15:e2001861. 10.1371/journal.pbio.2001861 28291793 PMC5349420

[B96] Rouzé R. Moné A. Delbac F. Belzunces L. Blot N. (2019). The honeybee gut microbiota is altered after chronic exposure to different families of insecticides and infection by *Nosema ceranae*. *Microbes Environ.* 34 226–233. 10.1264/jsme2.ME18169 31378758 PMC6759349

[B97] Royston J. P. (1982). An Extension of Shapiro and Wilk’s W Test for Normality to Large Samples. *Appl. Stat.* 31:115. 10.2307/2347973

[B98] Sammataro D. Finley J. Underwood R. (2008). Comparing oxalic acid and sucrocide treatments for *Varroa destructor* (Acari: *Varroidae*) control under desert conditions. *J. Econ. Entomol.* 101 1057–1061. 18767709 10.1603/0022-0493(2008)101[1057:coaast]2.0.co;2

[B99] Schneider S. Eisenhardt D. Rademacher E. (2012). Sublethal effects of oxalic acid on *Apis mellifera* (Hymenoptera: *Apidae*): changes in behaviour and longevity. *Apidologie* 43 218–225. 10.1007/s13592-011-0102-0

[B100] Shi J. Liao C. Wang Z. Zeng Z. Wu X. (2019). Effects of sublethal acetamiprid doses on the lifespan and memory-related characteristics of honey bee (*Apis mellifera*) workers. *Apidologie* 50 553–563. 10.1007/s13592-019-00669-w

[B101] Siede R. Faust L. Meixner M. D. Maus C. Grünewald B. Büchler R. (2017). Performance of honey bee colonies under a long-lasting dietary exposure to sublethal concentrations of the neonicotinoid insecticide thiacloprid. *Pest Manag. Sci.* 73 1334–1344. 10.1002/ps.4547 28168846 PMC5485166

[B102] Singh S. Singh S. K. Chowdhury I. Singh R. (2017). Understanding the Mechanism of Bacterial Biofilms Resistance to Antimicrobial Agents. *Open Microbiol. J.* 11 53–62. 10.2174/1874285801711010053 28553416 PMC5427689

[B103] Tarpy D. R. Mattila H. R. Newton I. L. G. (2015). Development of the honey bee gut microbiome throughout the queen-rearing process. *Appl. Environ. Microbiol*. 81, 3182–3191. 10.1128/AEM.00307-15 25724964 PMC4393441

[B104] Thany S. H. Bourdin C. M. Graton J. Laurent A. D. Mathé-Allainmat M. Lebreton J. (2015). Similar comparative low and high doses of deltamethrin and acetamiprid differently impair the retrieval of the proboscis extension reflex in the forager honeybee (*Apis mellifera*). *Insects* 6 805–814. 10.3390/insects6040805 26466901 PMC4693171

[B105] Therneau T. (2021). *A Package for Survival Analysis in R. R package version 3.2-11.* Available online at: https://CRAN.R-project.org/package=survival

[B106] Tian B. Fadhil N. H. Powell J. E. Kwong W. K. Moran N. A. (2012). Long-term exposure to antibiotics has caused accumulation of resistance determinants in the gut microbiota of honeybees. *mBio* 3 4–5. 10.1128/mBio.00377-12 23111871 PMC3487773

[B107] Tison L. Holtz S. Adeoye A. Kalkan Ö Irmisch N. S. Lehmann N. (2017). Effects of sublethal doses of thiacloprid and its formulation Calypso^®^ on the learning and memory performance of honey bees. *J. Exp. Biol.* 220 3695–3705. 10.1242/jeb.154518 28819056

[B108] Tosi S. Burgio G. Nieh J. C. (2017). A common neonicotinoid pesticide, thiamethoxam, impairs honey bee flight ability. *Sci. Rep.* 7:1201. 10.1038/s41598-017-01361-8 28446783 PMC5430654

[B109] US Department of Agriculture (USDA) (2016). *US Department of Agriculture.* Available online at: https://pubchem.ncbi.nlm.nih.gov/compound/Thiacloprid#section=USDA-Pesticide-Data-Program&fullscreen=true (accessed May 23, 2021).

[B110] US Department of Agriculture (USDA) (2021). *US Department of Agriculture.* Available online at: https://www.ars.usda.gov/oc/br/ccd/index/#:~:text=Logan%2C%20Utah-,U.S.%20Honey%20Bee%20Losses,Statistics%20Service%20(NASS)%20survey (accessed May 23, 2021).

[B111] US Environmental Protection Agency Office of Pesticide (2020). *Proposed Interim Registration Review Decision Case Number 7605 January 2020.* Washington: United States Environmental Protection Agency, 1–77.

[B112] vanEngelsdorp D. Traynor K. S. Andree M. Lichtenberg E. M. Chen Y. Saegerman C. (2017). Colony Collapse Disorder (CCD) and bee age impact honey bee pathophysiology. *PLoS One* 12:e0179535. 10.1371/journal.pone.0179535 28715431 PMC5513415

[B113] Vásquez A. Forsgren E. Fries I. Paxton R. J. Flaberg E. Szekely L. (2012). Symbionts as major modulators of insect health: lactic acid bacteria and honeybees. *PLoS One* 7:e33188. 10.1371/journal.pone.0033188 22427985 PMC3299755

[B114] Williamson S. M. Willis S. J. Wright G. A. (2014). Exposure to neonicotinoids influences the motor function of adult worker honeybees. *Ecotoxicology* 23 1409–1418. 10.1007/s10646-014-1283-x 25011924 PMC4165879

[B115] Zaworra M. Koehler H. Schneider J. Lagojda A. Nauen R. (2019). Pharmacokinetics of Three Neonicotinoid Insecticides upon Contact Exposure in the Western Honey Bee, *Apis mellifera* [Rapid-communication]. *Chem. Res. Toxicol.* 32 35–37. 10.1021/acs.chemrestox.8b00315 30525514

[B116] Zeileis A. Hothorn T. (2002). Diagnostic Checking in Regression Relationships. *R News* 2 7–10.

[B117] Zheng H. Nishida A. Kwong W. K. Koch H. Engel P. Steele M. I. (2016). Metabolism of toxic sugars by strains of the bee gut symbiont *Gilliamella apicola*. *mBio* 7 1–9. 10.1128/mBio.01326-16 27803186 PMC5090037

