# Supplementary material for: Resistance and Vulnerability of Honeybee (Apis mellifera) Gut Bacteria to Commonly Used Pesticides
Source: Front Microbiol. 2021 Sep 3;12:717990. doi: 10.3389/fmicb.2021.717990 (PMC8446526; doi:10.3389/fmicb.2021.717990)
Supplement: Supplementary file 1 [file Data_Sheet_1.zip › Supplementary Material.docx]

Supplementary Material

# Supplementary text

## Analyses including field controls

In addition to the experimental bees, we included 12 bees from each colony that had been frozen immediately after collection from the field (hereafter field controls). The processing of these samples, DNA extraction, 16S rRNA gene amplicon sequencing and statistical analysis, was the same as with the experimental bees (See Methods).

## Results

The field controls showed levels of alpha diversity comparable to the oxalic acid treated colonies (Tukey HSD: *p* = 1.0000; Figure S1A, Table S1), with reduced number of ASVs compared to controls. Including the field controls did not change the statistics previously reported without them. The field control replicated the oxalic acid treatment, being statistically different from the rest of the treatment groups (Tukey HSD: *p* < 0.0001, Tables S1-S2). Colonies 1 and 3 were very similar to each other and significantly different from colony 2 (Figure S1B; Tukey HSD: *p* > 0.05, Table S2), similar to when field controls were removed from the analysis (main text). The treatment effect was comparable across colonies, and the interaction of treatment and colony was not significant (F_8,167_ = 0.9585, *p* = 0.4703).

Beta diversity metrics were not greatly affected by the introduction of the field controls. Community composition was thus only to a small extent affected by treatment (Figure S1C) but mainly explained by colony differences (Figure S1D). PERMANOVA analysis confirmed that 24.5% of the variance was explained by colony, 8.6% by treatment, and 10.9% by the interaction between the two. Field controls were distinct from controls (F_1,66_ = 8.494; *p* = 0.0002) and comparable to oxalic acid.

The abundant genera remained similar across treatments, field controls and controls (Figure S2A), with colonies demonstrating variability as discussed in the main text (Figure S2B). However, we found five differentially abundant genera between field controls and controls (Figure S3A). *Bombella* increased in abundance in lab controls (t = 11.50; p < 0.0001), while *Bifidobacterium* (t = -8.570; *p* < 0.0001), *Gilliamella* (t = -8.074; p < 0.0001), *Lactobacillus* (t = -7.790; p < 0.0001) and *Frischella* (t = -3.743; *p* = 0.0052) all were relatively more abundant in the field controls than in the lab control. All of which, except *Frischella,* mirror the comparison between controls and oxalic acid treated honeybee microbiomes (Figure S3B). At ASV level, we found eight differentially abundant taxa between field controls and controls (Figure S3C). Increasing in abundance in the field control are a *Bombella intestini* (t = 13.82; *p* < 0.0001) and a *Lactobacillus* (t = 3.947; *p* = 0.0133) strain, as well as *L. kunkeei* (t = 11.36; *p* < 0.0001). The same three strains are negatively affected by oxalic acid compared to lab controls (Figure S3D). *L. kunkeei* was not completely absent in the field controls, rather in very small abundances, giving it the possibility to regrow after oxalic acid was removed from its environment (Figure S6). Conversely, a *Gilliamella* (t = -6.777; *p* < 0.0001), two *Lactobacillus* (t = -5.550; *p* = 0.0001; t = -5.029; *p* = 0.0010), and two *Bifidobacterium* strains (t = -5.307; *p* = 0.0005; t = -4.771; *p* = 0.0077) were more abundant in the field controls than lab controls*.* All genera and ASVs that are negatively affected by oxalic acid treatment increased in abundance during the seven days of our experiment (Figure S3), as we move from field colonies to lab controls: both comparisons effectively mirror each other.

## Discussion

We included field controls that were not included in the focal experiment and found them to be strikingly similar in composition and richness to oxalic acid treated sub-colonies. To our knowledge, the colonies included in our experiment were exposed to oxalic acid 8 months and 9 days before our sampling. After our sampling, we found out other beehives in the vicinity (~40m) were also treated during the winter before our sampling. Given oxalic acid has been reported to remain within beehive material for up to 6 months (Rademacher et al., 2017), the most parsimonious explanation for the similarities between field control and oxalic acid treated bee microbiomes would be that the field colonies were exposed to oxalic acid. We cannot confirm this, but the microbiome results are compelling.

On the other hand, we saw that *Bombella intestini*, *Lactobacillus kunkeei*, and another *Lactobacillus* significantly increase, and decrease, in abundances between field controls and lab controls, and between lab controls and oxalic acid treatment, respectively. The strikingly short duration of our experiment (seven days), during which these microbes regrew from being locally extinct (or below detection threshold) in most bees, suggest ample capacity of the microbiome to stabilize itself under physiological conditions. Whether there were strain-specific oxalic-acid driven extinctions that could not re-emerge once oxalic acid presence was removed in our laboratory experiment, we cannot assess.

Either way, we consider the results presented in the main text to be robust, as the comparison between lab controls and oxalic acid-treated honeybee controls is a direct comparison unaffected by previous exposure to oxalic acid in the field.

## References

Rademacher, E., Harz, M., & Schneider, S. (2017). Effects of oxalic acid on apis mellifera (Hymenoptera: Apidae). *Insects*, *8*(3). https://doi.org/10.3390/insects8030084

# Supplementary Figures and Tables

## Supplementary Figures

**Figure S1.** Alpha diversity and beta diversity plots that include field controls. **(A)** and **(B)** give alpha diversity measurements as the number of ASVs per treatment, panels **(C)** and **(D)** reference beta diversity measurements. Colours indicate pesticide treatment in panels **(A)** through **(C)**. (**A)** Number of ASVs across treatments. **(B)** Number of ASVs across treatments and colonies. Boxplots represent the first and third quartiles of the number of ASVs observed per treatment, the horizontal line represents the median, whiskers extend 1.5 interquartile ranges and dots represent outliers. In panel **(B)**, the letters represent the statistical dissimilarities across groups according to Tukey HSD test (*p* < 0.05; Tables S4, S5). **(C)** Non-metric multidimensional scaling (NMDS) analysis, based on the Bray-Curtis distances based on treatment (colours by pesticides, as in **(A)**). **(D)** NMDS plot ordination based on colony. There is a clearer clustering in the plot ordination based on colony, supported by the PERMANOVA Adonis test, that indicates that colony explained 24.5% (*p* < 0.0001) and treatment 8.6% (*p* < 0.0001) of the variation in community structure.

**Figure S2.** Relative abundances of the 15 most abundant genera across treatments. **A**. Mean relative abundances of the 15 most abundant genera for each treatment. **B**. Relative abundance of the 15 most abundant genera showing the variability of each sample.

**Figure S3.** ALDEx2 results of differentially abundant (*p* < 0.05) genera and ASVs between treatments. **(A)** Comparison of the genera that vary in the field control compared to the laboratory control. Positive values indicate increased relative abundance in controls; negative values indicate increased relative abundance in field controls. **(B)** Comparison of the genera that vary in the laboratory control compared to the oxalic acid treatment. Positive values indicate increased relative abundance in oxalic acid treatment; negative values indicate increased relative abundance in controls. **(C)** Comparison of ASVs in the field control compared to the laboratory control. Positive values indicate increased relative abundance in laboratory controls; negative values indicate increased relative abundance in field controls. **(D)** Comparison of the ASVs that vary in the laboratory control compared to the oxalic acid treatment. Positive values indicate increased relative abundance in oxalic acid treatment; negative values indicate increased relative abundance in controls.


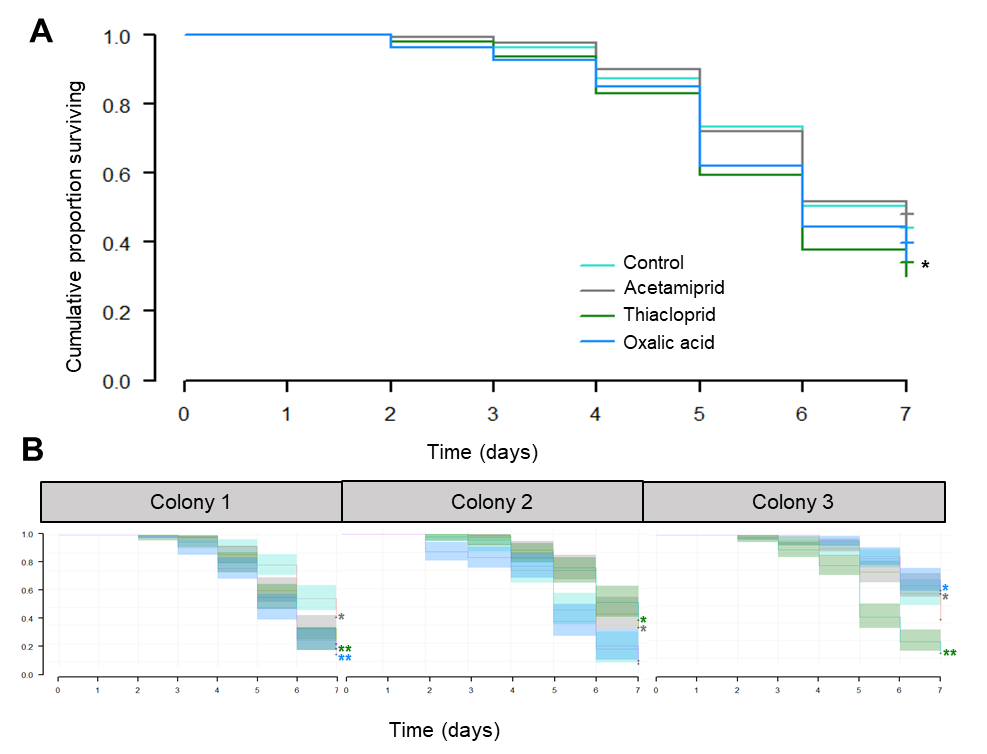
**Figure S4.** Detailed Cox proportional hazard regression curve of mortality. **(A)** Cox proportional hazard regression curve of mortality throughout the experiment across all three colonies. Treatment thiacloprid significantly reduced survival (*p* < 0.05). **(B)** Cox proportional hazard regression curve of mortality throughout the experiment for each of the colonies, with coloured shading representing the 95% confidence intervals.


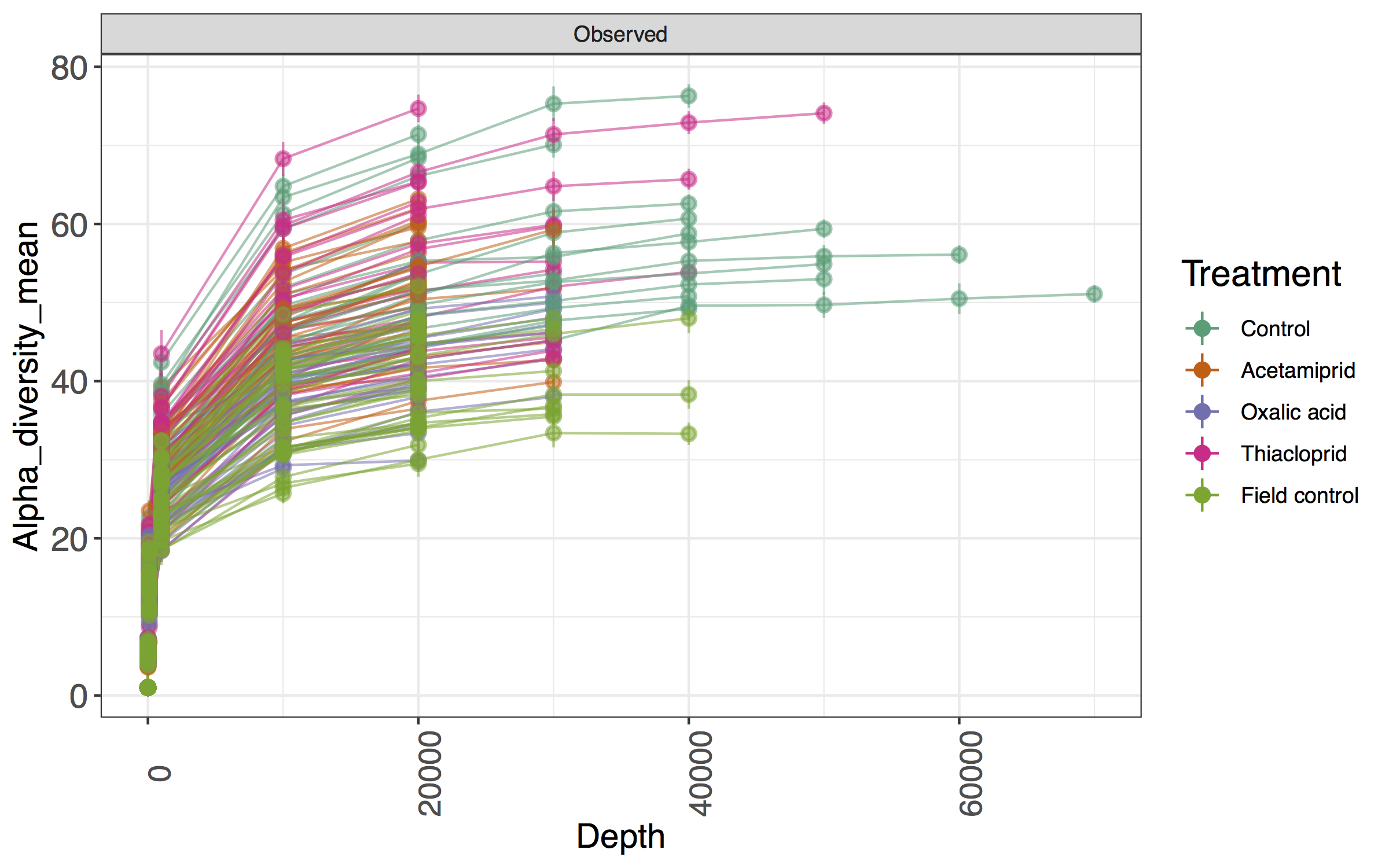


**Figure S5**. Rarefaction curves.


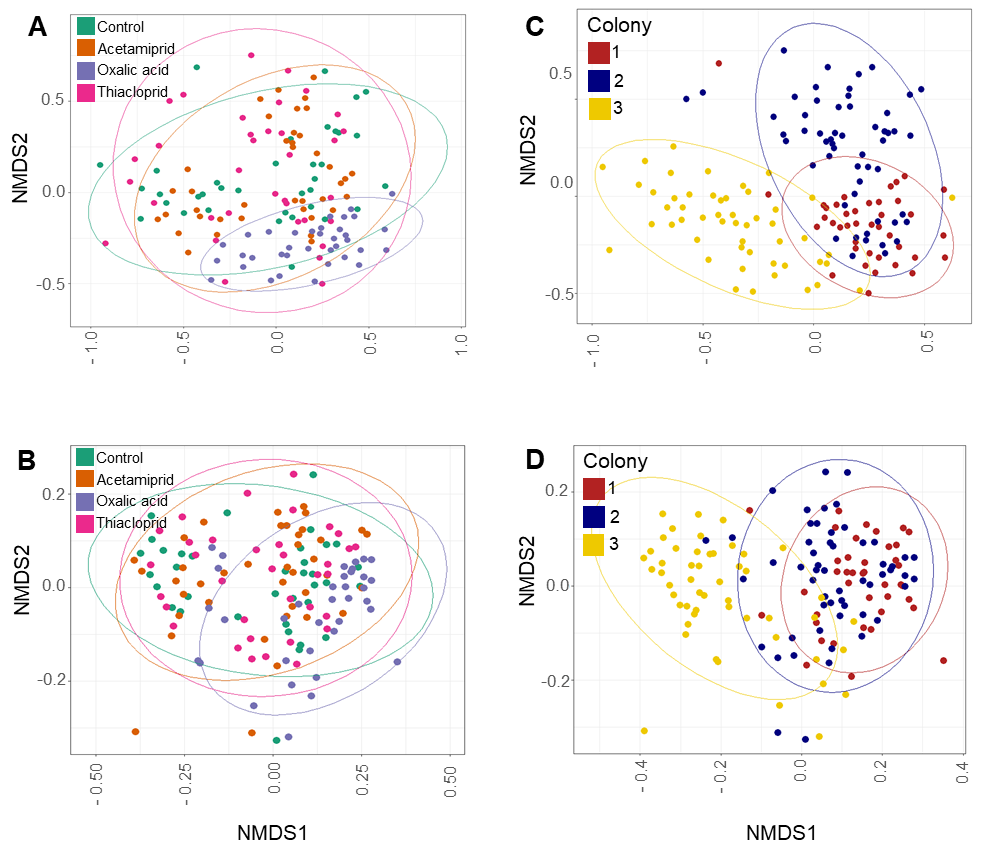


**Figure S6**.  Different beta diversity estimates. Panels **A** and **C** reference beta diversity Jaccard measurements, panels **B** and **D** reference beta diversity Unifrac measurements. **A**. Nonmetric multidimensional scaling (NMDS) analysis of the samples based on Jaccard distances; plot ordination based on treatment. **B**. Nonmetric multidimensional scaling (NMDS) analysis of the samples based on Jaccard distances; plot ordination based on colony. PERMANOVA analysis based on Jaccard distances indicated that 19.7% of the variability was explained by colony, 5.3% by treatment, and 8.7% by their interaction. According to Jaccard distances, oxalic acid treatment generates the most distinct microbiomes (F_1,66_ = 5.557; *p* < 0.0001), followed by acetamiprid (F_1,69_ = 2.151; *p* = 0.0278) and thiacloprid (F_1,70_ = 1.844; *p* = 0.0413). **C**. Nonmetric multidimensional scaling (NMDS) analysis of the samples based on Unifrac distances; plot ordination based on treatment. **D**. Nonmetric multidimensional scaling (NMDS) analysis of the samples based on Unifrac distances; plot ordination based on colony. PERMANOVA analysis based on Unifrac distances indicated that 38.8% of the variability was explained by colony, 8.6% by treatment, and 8.6% by their interaction. According to Unifrac distances, oxalic acid treatment generates the most distinct microbiomes (F_1,66_ = 17.6; *p* < 0.0001), followed by acetamiprid (F_1,69_ = 0.769; *p* = 0.473) and thiacloprid (F_1,70_ = 0.348; *p* = 0.827).

**Figure S7**. Abundance of ASV_19, identified as *Lactobacillus kunkeei*, across treatments (top x axis) and colonies 1-3 (y-axis).

## Supplementary Tables

**Table S1.** Pairwise comparisons of alpha diversity across treatments including the field controls. The statistical test used was the Tukey Honestly Significant Difference (HSD) post hoc multiple comparisons test for alpha diversity.

| **Comparison** | ***p*-value** | **Comparison** | ***p*-value** |
| --- | --- | --- | --- |
| Acetamiprid - Control | 0.0850 | Oxalic acid – Acetamiprid | <0.0001 |
| Oxalic acid - Control | <0.0001 | Thiacloprid - Acetamiprid | 0.9601 |
| Thiacloprid – Control | 0.3699 | Thiacloprid – Oxalic acid | <0.0001 |
| Control – Field control | <0.0001 | Oxalic acid – Field control | 1 |
| Acetamiprid – Field control | <0.0001 | Thiacloprid – Field control | <0.0001 |

**Table S2.** Pairwise comparisons of alpha diversity across colonies, including field controls. The statistical test used was Tukey Honestly Significant Difference (HSD) post hoc multiple comparisons test for alpha diversity.

| **Colony 1** | | | |
| --- | --- | --- | --- |
| **Comparison** | ***p*-value** | **Comparison** | ***p*-value** |
| Acetamiprid - Control | 0.9999 | Oxalic acid – Acetamiprid | 0.0184 |
| Oxalic acid - Control | 0.0042 | Thiacloprid - Acetamiprid | 1.0000 |
| Thiacloprid – Control | 0.9999 | Thiacloprid – Oxalic acid | 0.0618 |
| Control – Field control | 0.0010 | Oxalic acid – Field control | 1.0000 |
| Acetamiprid – Field control | 0.0041 | Thiacloprid – Field control | 0.0239 |
| **Colony 2** | | | |
| Acetamiprid - Control | 0.9839 | Oxalic acid – Acetamiprid | 0.0072 |
| Oxalic acid - Control | <0.0001 | Thiacloprid - Acetamiprid | 0.9882 |
| Thiacloprid – Control | 1.0000 | Thiacloprid – Oxalic acid | <0.0001 |
| Control – Field control | <0.0001 | Oxalic acid – Field control | 1.0000 |
| Acetamiprid – Field control | 0.0021 | Thiacloprid – Field control | <0.0001 |
| **Colony 3** | | | |
| Acetamiprid - Control | 0.2852 | Oxalic acid – Acetamiprid | 0.0036 |
| Oxalic acid - Control | <0.0001 | Thiacloprid - Acetamiprid | 0.9999 |
| Thiacloprid – Control | 0.0668 | Thiacloprid – Oxalic acid | 0.0794 |
| Control – Field control | <0.0001 | Oxalic acid – Field control | 1.0000 |
| Acetamiprid – Field control | 0.0048 | Thiacloprid – Field control | 0.0883 |

**Table S3.** Mortality across the *in vivo* experiment. Each of the rows represent each of the bees in our experiment. Replicate refers to the sub-colony classification, for each colony, each treatment was replicated in 5 sub-colonies. Censored column references a death event happening (honeybee death: TRUE) or not (honeybee survival: FALSE) (separate excel file).

**Table S4.** Raw data for the presence/absence of consumption and estimate volume of sugar water consumption across treatments (separate excel file).

**Table S5.** Volume of pesticide or control consumed per bee over day one through four of the experiment, accounting for mortality. Days 5 through 7 were not assessed as consumption was minimal and not reliably quantified. The average and standard deviation across all sub-colonies is provided (n_day1_ = 75, n_day2_ = 72, n_day3_ = 53, n_day4_ = 22).

| Day of Treatment | Control | Acetamiprid | Thiacloprid | Oxalic acid |
| --- | --- | --- | --- | --- |
| **Day 1** | 0.0747±0.0192 | 0.0707±0.0212 | 0.0720±0.0237 | 0.0680±0.0237 |
| **Day 2** | 0.0565±0.0223 | 0.0704±0.0217 | 0.0736±0.0288 | 0.0526±0.0283 |
| **Day 3** | 0.0508±0.0313 | 0.0619±0.0293 | 0.0759±0.0309 | 0.0316±0.0393 |
| **Day 4** | 0.0377±0.0154 | 0.0138±0.0215 | 0.0542±0.0498 | 0.0218±0.0301 |

**Table S6.** Average volume of pesticide or control consumed per bee per colony over day one through four of the experiment, accounting for mortality. Averages are presented across sub-colonies along with standard deviation when possible.

| Colony | Day of treatment | Control | Acetamiprid | Thiacloprid | Oxalic acid |
| --- | --- | --- | --- | --- | --- |
| **Colony 1** | **Day 1** | 0.0640±0.0167 | 0.0560±0.0219 | 0.0560±0.0167 | 0.0600±0.0245 |
|  | **Day 2** | 0.0480±0.0110 | 0.0540±0.0191 | 0.0738±0.0113 | 0.0646±0.0175 |
|  | **Day 3** | 0.0600±0.0283 | 0.0510±0.0200 | 0.0420±0.0000 | 0.0498±0.0530 |
|  | **Day 4** | 0.0200 | 0.0215±0.0304 | 0.0000 | 0.0067±0.0115 |
| **Colony 2** | **Day 1** | 0.0720±0.0228 | 0.0680±0.0178 | 0.0720±0.0303 | 0.0680±0.02280 |
|  | **Day 2** | 0.0534±0.0350 | 0.0640±0.0167 | 0.0623±0.0461 | 0.0535±0.0420 |
|  | **Day 3** | 0.0433±0.0333 | 0.0466±0.0416 | 0.0747±0.0254 | 0.0130±0.0291 |
|  | **Day 4** | NA | 0.0000±0.0000 | 0.0870 | 0.0320±0.0288 |
| **Colony 3** | **Day 1** | 0.0880±0.0110 | 0.0880±0.0110 | 0.0880±0.0179 | 0.0760±0.0261 |
|  | **Day 2** | 0.0680±0.0110 | 0.0890±0.0160 | 0.0826±0.0273 | 0.0400±0.0245 |
|  | **Day 3** | 0.0538±0.0376 | 0.0843±0.0154 | 0.0938±0.0303 | 0.0320±0.0303 |
|  | **Day 4** | 0.0465±0.0021 | 0.0200±0.0283 | 0.0650±0.0566 | 0.0267±0.0462 |

**Table S7.** Pairwise comparisons of ASV richness across treatments using the Tukey Honestly Significant Difference (HSD) post hoc test for multiple comparisons.

| **Comparison** | ***p*** | **Comparison** | ***p*** |
| --- | --- | --- | --- |
| Acetamiprid - Control | 0.0522 | Oxalic acid – Acetamiprid | <0.0001 |
| Oxalic acid - Control | <0.0001 | Thiacloprid - Acetamiprid | 0.9002 |
| Thiacloprid – Control | 0.2602 | Thiacloprid – Oxalic acid | <0.0001 |

**Table S8.** Pairwise comparisons of ASV richness across colonies using the Tukey Honestly Significant Difference (HSD) post hoc test for multiple comparisons.

| **Comparison** | ***p*** |
| --- | --- |
| Colony 1 – Colony 2 | < 0.0001 |
| Colony 1 – Colony 3 | 0.9683 |
| Colony 2 – Colony 3 | < 0.0001 |

**Table S9**. Absolute number of sequences per ASV for the full dataset (including field controls). Each of the rows represent each of the bees that were dissected for our gut microbiome study. Replicate refers to the sub-colony classification, for each colony, each treatment was replicated in five sub-colonies (separate excel file).
